# Enhancing Oral Bioavailability of Poorly Water-Soluble Natural Products via Lipid–Drug Conjugates

**DOI:** 10.3390/pharmaceutics18070899

**Published:** 2026-07-22

**Authors:** Xiaoli Zou, Bin Zhang, Lianghe Mei, Sifei Han, Kaixian Chen

**Affiliations:** 1 School of Chinese Materia Medica, Nanjing University of Chinese Medicine, Nanjing 210023, China; zouxiaoli@susimm.cn (X.Z.); zhangbin@susimm.cn (B.Z.); 2 Shanghai Institute of Materia Medica, Chinese Academy of Sciences, Shanghai 201203, China; 3 Suzhou Institute of Materia Medica, Suzhou 215123, China; meilianghe@susimm.cn; 4 School of Pharmacy, China Pharmaceutical University, 24 Tongjia Lane, Nanjing 210009, China

**Keywords:** lipid–drug conjugates, oral exposure, natural products, pharmacokinetics, lymphatic transport

## Abstract

**Background:** The therapeutic potential of many natural products, including curcumin (CUR), betulinic acid (BA), and oleanolic acid (OA), is limited by poor oral exposure caused by low aqueous solubility, metabolic instability, and/or first-pass metabolism. Lipid–drug conjugate (LDC) strategies that mimic endogenous dietary lipid processing may provide a useful approach for improving oral absorption and lymphatic transport. **Methods:** A 1,3-diolein-based lipidic promoiety (IN-4) was synthesized and conjugated to curcumin, betulinic acid, and oleanolic acid to generate three representative LDCs: CUR-PRO, BA-PRO, and OA-PRO. Their oral pharmacokinetic behavior was evaluated in rats. For CUR-PRO, matched-vehicle comparisons across three oral vehicles were performed, together with mesenteric lymph duct cannulation and in vitro stability/conversion studies in simulated gastrointestinal media, rat liver microsomes, and rat plasma. **Results:** All three prodrugs were successfully synthesized and showed improved systemic exposure to the corresponding parent-drug-related analytes under the tested conditions. For CUR-PRO, dose-normalized AUC_0-last_ of released curcumin was markedly higher than direct curcumin administration across all three vehicles (increases of 15.0-, 70.9-, and 54.3-fold), and intact CUR-PRO was also detected in plasma. Mesenteric lymph sampling showed that CUR-PRO dosing, but not free-curcumin dosing, generated detectable curcumin-related signals under the present analytical conditions. In vitro, no free curcumin was detected during CUR-PRO incubation in enzyme-free simulated gastrointestinal media; CUR-PRO underwent rapid depletion in pancreatic-lipase-supplemented medium, showed greater microsomal stability than curcumin, and displayed plasma conversion that was markedly accelerated by exogenous LPL. BA-PRO and OA-PRO also increased systemic exposure of their released parent drugs, with 16.0- and 38.4-fold dose-normalized AUC_0-last_ increases, respectively. **Conclusions:** These findings provide proof-of-concept evidence that 1,3-diolein-based lipidation can improve the oral exposure of selected poorly water-soluble natural products. The lymphatic transport data provide qualitative evidence supporting lymphatic access of CUR-PRO, although the quantitative contribution of this pathway to the overall exposure increase remains to be established.

## 1. Introduction

Natural products remain an important source of therapeutic leads and drug candidates because of their structural diversity and broad pharmacological activities [1,2,3,4,5]. However, the clinical translation of many natural products is frequently limited by poor oral bioavailability, which is often associated with low aqueous solubility, limited intestinal absorption, chemical or metabolic instability, and extensive first-pass metabolism [6,7,8]. Curcumin, betulinic acid, and oleanolic acid are representative examples of bioactive natural products with promising pharmacological activities but suboptimal oral pharmacokinetic profiles [9,10,11]. Strategies capable of improving their systemic exposure are therefore important for advancing their translational potential.

A variety of formulation and drug-delivery approaches have been investigated to overcome these limitations, including nanocarriers, lipid-based systems, surfactant-based solubilization, solid dispersions, particle engineering, salt or polymorph screening, and chemical modification [12,13,14,15,16]. Among these approaches, intestinal lymphatic drug delivery has attracted increasing interest because it can promote the absorption of highly lipophilic compounds through lipid-processing pathways and may reduce the impact of hepatic first-pass metabolism [17,18].

The intestinal lymphatic pathway is closely linked to dietary lipid digestion, absorption, and chylomicron assembly. Following intestinal uptake, long-chain lipids are re-esterified within enterocytes and incorporated into chylomicrons, which are subsequently transported through the mesenteric lymph before entering the systemic circulation [19,20,21,22,23,24]. Drug-delivery systems that interact with this endogenous pathway may therefore enhance oral exposure and alter tissue distribution profiles for appropriately designed lipophilic molecules.

Lipid-based formulations can facilitate lymphatic uptake by increasing solubilization in the intestinal lumen and promoting association with lipid digestion products. In parallel, covalent lipidation has emerged as a prodrug-based strategy in which drug molecules are chemically conjugated to fatty acid, glyceride, or other lipid motifs [25,26,27]. Compared with simple physical solubilization, lipid–drug conjugates can be designed to mimic endogenous lipid substrates and thereby engage specific enzymatic and trafficking processes involved in intestinal lipid absorption.

Triglyceride- and glyceride-mimetic prodrugs are particularly attractive because they can be processed in a manner analogous to dietary triglycerides. Previous studies have shown that appropriately designed glyceride-mimetic prodrugs can promote intestinal lymphatic transport, reduce direct portal exposure, and increase systemic oral exposure of selected model drugs. For example, triglyceride-based prodrugs of buprenorphine incorporating self-immolative linkers achieved up to 45% lymphatic delivery after intestinal lipolysis, compared with less than 0.1% for free buprenorphine, and resulted in a 22-fold increase in oral bioavailability [28]. Similarly, 1,3-diacylglycerol-based testosterone prodrugs containing self-immolative spacers redirected drug transport from the portal vein to the intestinal lymphatic pathway, leading to up to a 90-fold increase in oral plasma exposure relative to a conventional prodrug [29]. These findings provide a mechanistic basis for applying triglyceride-mimetic lipidation to poorly absorbed natural products.

The clinical translation of lymph-directed prodrug strategies has also shown encouraging progress. GlyphAllo™ (SPT-300, formerly LYT-300), an oral allopregnanolone prodrug developed using lymphatic-targeted prodrug technology, has demonstrated oral bioavailability, tolerability, and GABA_A receptor target engagement in early clinical studies and has advanced into a Phase 2b clinical trial for major depressive disorder with or without anxious distress [30,31]. More recently, preclinical and first-in-human data for GlyphAllo were reported, further supporting the clinical feasibility of triglyceride-mimetic prodrug design for achieving therapeutically relevant systemic exposure after oral dosing [32]. These advances provide a translational framework for the development of lymph-directed lipidized prodrugs and support further exploration of this strategy for improving the oral delivery of poorly absorbed natural products.

Despite these advances, the applicability of triglyceride-mimetic prodrug strategies to selected poorly water-soluble natural products remains insufficiently explored. In particular, it remains unclear whether representative natural products such as curcumin, betulinic acid, and oleanolic acid can be rationally converted into triglyceride-mimetic lipid–drug conjugate (LDC) prodrugs and subsequently generate measurable systemic exposure to their released parent drugs in vivo. This gap is especially relevant because natural products differ substantially in functional groups, lipophilicity, and metabolic liabilities, which may influence prodrug conversion, intestinal lipid processing, lymphatic transport, and systemic parent-drug release.

**Figure 1:**
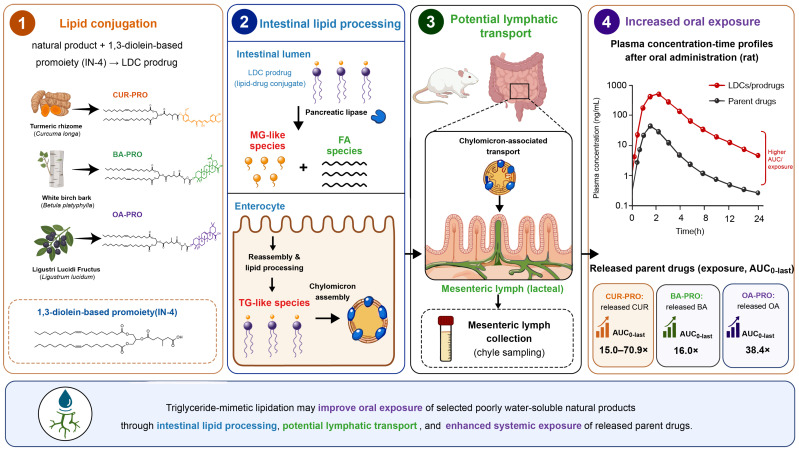
Four-step conceptual overview of the 1,3-diolein-based lipid–drug conjugate strategy and the corresponding experimental framework. ① Lipid conjugation: Curcumin, betulinic acid, and oleanolic acid were conjugated to the common 1,3-diolein-based promoiety IN-4 to generate CUR-PRO, BA-PRO, and OA-PRO. ② Intestinal lipid processing: In the intestinal lumen, pancreatic lipase-mediated digestion is proposed to generate monoglyceride-like (MG-like) and fatty-acid (FA) species, followed by enterocyte uptake, reassembly into triglyceride-like (TG-like) species, and chylomicron assembly. ③ Potential lymphatic transport: Chylomicron-associated prodrug species may access mesenteric lymph, as assessed by lymph collection in rats. ④ Increased oral exposure: Increased systemic exposure to released parent drugs was observed after oral prodrug administration. The AUC_0-last_ fold increases shown are dose-normalized exposure ratios under the tested conditions. The scheme represents a working hypothesis and does not imply that lymphatic transport is the sole determinant of the observed pharmacokinetic enhancement.

In this study, we developed a 1,3-diolein-based LDC strategy to improve the oral exposure of selected poorly water-soluble natural products. A common lipidic promoiety (IN-4) was synthesized and conjugated to curcumin, betulinic acid, and oleanolic acid to generate three representative prodrugs: CUR-PRO, BA-PRO, and OA-PRO. Figure 1 summarizes the study in a four-step experimental and conceptual sequence: (1) lipid conjugation of the selected natural products to the common 1,3-diolein-based promoiety; (2) proposed pancreatic lipase-mediated intestinal processing, followed by enterocyte uptake, reassembly into triglyceride-like species, and chylomicron assembly; (3) potential chylomicron-associated access to mesenteric lymph, evaluated by lymph collection in rats; and (4) increased systemic exposure to the released parent drugs after oral administration. This scheme is presented as a working hypothesis and does not imply that lymphatic transport is the sole determinant of the observed pharmacokinetic changes. The prodrugs were evaluated using exploratory rat pharmacokinetic studies, with detailed matched-vehicle analysis for CUR-PRO. Mesenteric lymph duct cannulation and complementary in vitro stability/conversion studies were further conducted to examine lymphatic access and enzymatic processing of CUR-PRO. Together, these studies provide an early-stage, multi-faceted proof-of-concept assessment of triglyceride-mimetic lipidation for selected poorly water-soluble natural products, while the relative contributions of dissolution or dispersion, permeability, metabolism, disposition, prodrug conversion, and lymphatic transport remain to be resolved. The principal novelty of this work is the integrated evaluation of a common 1,3-diolein-based promoiety across three selected natural products spanning two structural classes, combining cross-compound exposure assessment with matched-vehicle pharmacokinetics, direct mesenteric lymph sampling, and complementary enzymatic studies.

## 2. Materials and Methods

### 2.1. Materials

Curcumin (purity ≥ 98%), betulinic acid (purity ≥ 98%), and oleanolic acid (purity ≥ 98%) were purchased from Macklin (Shanghai, China). Ethyl 4-oxopentanoate (IN-4a), tert-butyl (dimethoxyphosphoryl)acetate, and 1,3-dioleoylglycerol (1,3-diolein, IN-2b) were obtained from Chinasun Specialty Products Co., Ltd. (Changshu, China) 1-(3-Dimethylaminopropyl)-3-ethylcarbodiimide hydrochloride (EDC·HCl), 4-dimethylaminopyridine (DMAP), N,N-diisopropylethylamine (DIPEA), 1-chloroethyl chloroformate (CEOC), and pyridine were obtained from Shanghai Bepharm Science & Technology Co., Ltd. (Shanghai, China) Palladium on carbon (Pd/C, 10 wt%) and tetrakis(triphenylphosphine)palladium(0) [Pd(PPh3)4] were purchased from J&K Chemical Ltd. (Shanghai, China).

Sodium hydride (NaH, 60% dispersion in mineral oil), potassium carbonate (K_2_CO_3_), cesium carbonate (Cs_2_CO_3_), sodium hydroxide (NaOH), hydrochloric acid (HCl), trifluoroacetic acid (TFA), 1,3-dimethylbarbituric acid, and tetrabutylammonium iodide (TBAI), anhydrous sodium sulfate (Na_2_SO_4_), anhydrous tetrahydrofuran (THF), anhydrous dichloromethane (DCM), N,N-dimethylformamide (DMF), toluene, methanol, ethyl acetate, and petroleum ether (boiling range, 60–90 °C) were obtained from Chinasun Specialty Products Co., Ltd. (Changshu, China) Allyl bromide was purchased from Aladdin (Shanghai, China).

Polyethylene glycol 400 (PEG 400), diethylene glycol monoethyl ether (Transcutol^®^ HP), rice bran oil, Lipoid^®^ E 80, Tween 80, olive oil, oleic acid, ethanol, sodium carboxymethyl cellulose (CMC-Na), and propylene glycol were provided by Gattefossé (Lyon, France). Solutol^®^ HS 15 was purchased from Merck (Darmstadt, Germany), and sodium chloride for the preparation of 0.9% saline was obtained from Shaanxi Shengao Animal Pharmaceutical Co., Ltd. (Xi’an, Shaanxi, China) Polyethylene cannulation tubing (PE-50) was purchased from Instech Laboratories (Plymouth Meeting, PA, USA), and 5% glucose injection was obtained from Sichuan Kelun Pharmaceutical Co., Ltd. (Chengdu, China).

EDTA-K2 anticoagulant blood collection tubes were obtained from Nantong HaiRui Experimental Equipment Co., Ltd. (Nantong, China) Lipoprotein lipase (LPL) was purchased from Sigma-Aldrich (St. Louis, MO, USA), and porcine pancreatic lipase was purchased from Shanghai Yuanye Bio-Technology Co., Ltd. (Shanghai, China) Fasted-state simulated intestinal fluid (FaSSIF), fed-state simulated intestinal fluid (FeSSIF), and fasted-state simulated gastric fluid (FaSSGF) powders were obtained from Biorelevant Limited (London, UK). Rat liver microsomes were purchased from IPHASE Bioscience Co., Ltd. (Kunshan, China) Sodium hydroxide, hydrochloric acid, sodium chloride, acetic acid, and sodium dihydrogen phosphate were purchased from Chinasun Specialty Products Co., Ltd. (Changshu, China) Tris-maleate and verapamil were purchased from Shanghai Macklin Biochemical Co., Ltd. (Shanghai, China), and calcium chloride monohydrate was obtained from Anhui Zesheng Science & Technology Co., Ltd. (Anqing, China) NADPH was purchased from Beijing Solarbio Science & Technology Co., Ltd. (Beijing, China).

For HPLC analysis, HPLC-grade acetonitrile and methanol were obtained from TEDIA (Fairfield, OH, USA). For LC–MS/MS bioanalysis, acetonitrile (LC–MS grade, TEDIA (Fairfield, OH, USA)), formic acid (LC–MS grade, Adamas-beta (Shanghai, China)), ammonium acetate (LC–MS grade, MREDA (Beijing, China)), and LC–MS-grade water were used. Deuterated solvents for NMR spectroscopy (CDCl3 and DMSO-d6) were obtained from TCI (Tokyo, Japan).

Unless otherwise stated, all reagents and solvents were of analytical or synthetic grade and were used as received without further purification.

### 2.2. Preparation of Prodrugs

#### 2.2.1. Preparationof the 1,3-Diolein-Based Lipid Promoiety (IN-4)

Synthesis of tert-butyl 6-ethyl (E)-3-methylhex-2-enedioate (IN-4b): To a solution of tert-butyl (dimethoxyphosphoryl)acetate (18.60 g, 82.91 mmol) in anhydrous tetrahydrofuran (THF, 100 mL) at 0 °C under a nitrogen atmosphere was added sodium hydride (3.60 g, 90.0 mmol, 60% dispersion in mineral oil) portionwise. The reaction mixture was stirred at 0 °C for 1 h to generate the phosphonate anion, after which ethyl 4-oxopentanoate (IN-4a, 10.00 g, 69.30 mmol) was added dropwise. The reaction was continued at 0 °C for 2 h and monitored by TLC (petroleum ether/ethyl acetate = 10:1). After complete consumption of the starting material (Rf = 0.3), the reaction was quenched with water (100 mL) and extracted with ethyl acetate (2 × 200 mL). The combined organic layers were dried over anhydrous Na_2_SO_4_, filtered, and concentrated under reduced pressure. The crude residue was purified by flash column chromatography (petroleum ether/ethyl acetate = 30:1) to afford IN-4b as a colorless oil (12.91 g, 77.3%). ^1^H NMR (400 MHz, CDCl_3_) δ 5.59 (s, 1H), 4.14 (q, *J* = 7.2 Hz, 2H), 2.53–2.40 (m, 4H), 2.13 (s, 3H), 1.47 (s, 9H), 1.26 (t, *J* = 7.2 Hz, 3H).

Synthesis of tert-butyl 6-ethyl 3-methylhexanedioate (IN-4c): Compound IN-4b (5.01 g, 20.7 mmol) was dissolved in methanol (50 mL). Palladium on carbon (10 wt%, 500 mg) was added, and the reaction vessel was evacuated and backfilled with hydrogen for three cycles. The mixture was stirred under a H_2_ atmosphere at 25 °C for 36 h and monitored by TLC (petroleum ether/ethyl acetate = 10:1) until the starting material (Rf = 0.3) was completely consumed. The catalyst was removed by filtration through a pad of Celite^®^, and the filter cake was rinsed with methanol (2 × 10 mL). The filtrate was concentrated in vacuo to give crude IN-4c as a colorless oil (5.00 g), which was used directly in the next step. ^1^H NMR (400 MHz, CDCl_3_) δ 4.13 (q, *J* = 7.2 Hz, 2H), 2.42–2.26 (m, 2H), 2.22 (dd, *J* = 14.8, 6.0 Hz, 1H), 2.05 (dd, *J* = 14.4, 8.0 Hz, 1H), 1.99–1.89 (m, 1H), 1.73–1.64 (m, 1H), 1.58–1.49 (m, 1H), 1.45 (s, 9H), 1.25 (t, *J* = 7.2 Hz, 3H), 0.95 (d, *J* = 6.4 Hz, 3H).

Synthesis of 6-(tert-butoxy)-4-methyl-6-oxohexanoic acid (IN-4d): Crude IN-4c (5.00 g, theoretical) was dissolved in a mixture of THF and water (20 mL/20 mL). Solid sodium hydroxide (1.20 g, 30.0 mmol) was added, and the resulting mixture was stirred vigorously at 25 °C for 18 h and monitored by TLC (petroleum ether/ethyl acetate = 10:1). After complete hydrolysis, the mixture was acidified to pH 5–6 with 3 M aqueous HCl and extracted with ethyl acetate (2 × 100 mL). The combined organic phases were dried over anhydrous Na_2_SO_4_ and concentrated to afford monoacid IN-4d as a colorless oil (4.01 g, 89.6% yield over two steps from IN-4b). ^1^H NMR (400 MHz, CDCl_3_) δ 2.49–2.29 (m, 2H), 2.22 (dd, *J* = 14.4, 6.0 Hz, 1H), 2.07 (dd, *J* = 14.4, 7.8 Hz, 1H), 2.01–1.92 (m, 1H), 1.77–1.65 (m, 1H), 1.61–1.48 (m, 1H), 1.45 (s, 9H), 0.96 (d, *J* = 6.8 Hz, 3H).

Synthesis of 1-tert-butyl 6-((1,3-bis(oleoyloxy)propan-2-yl) oxy)-3-methylhexanedioate (IN-4e): To a stirred solution of IN-4d (2.00 g, 9.23 mmol) in anhydrous dichloromethane (DCM, 10 mL) were added 1,3-dioleoylglycerol (IN-2b, 1.91 g, 3.07 mmol), 1-(3-dimethylaminopropyl)-3-ethylcarbodiimide hydrochloride (EDC·HCl, 1.17 g, 6.12 mmol), and 4-dimethylaminopyridine (DMAP, 374 mg, 3.06 mmol) sequentially at 25 °C under a nitrogen atmosphere. The mixture was stirred at 25 °C for 18 h and monitored by TLC (petroleum ether/ethyl acetate = 4:1). After completion, the reaction was quenched with water (20 mL) and extracted with DCM (2 × 20 mL). The combined organic extracts were dried over anhydrous Na_2_SO_4_, concentrated, and purified by flash column chromatography (petroleum ether/ethyl acetate = 10:1) to furnish IN-4e as a colorless oil (1.27 g, 50.6%). ^1^H NMR (400 MHz, CDCl_3_) δ 5.42–5.21 (m, 5H), 4.29 (dd, *J* = 12.0, 4.4 Hz, 2H), 4.14 (dd, *J* = 12.0, 6.0 Hz, 2H), 2.38–2.17 (m, 8H), 2.07–1.97 (m, 8H), 1.66–1.57 (m, 7H), 1.44 (s, 9H), 1.32–1.25 (m, 40H), 0.88–0.87 (m, 6H).

Synthesis of 6-((1,3-bis(oleoyloxy)propan-2-yl) oxy)-3-methyl-6-oxohexanoic acid (IN-4): Compound IN-4e (1.27 g, 1.55 mmol) was dissolved in anhydrous DCM (10 mL). Trifluoroacetic acid (5 mL) was added dropwise at 25 °C, and the mixture was stirred at 25 °C for 18 h and monitored by TLC (petroleum ether/ethyl acetate = 4:1). After complete deprotection of the tert-butyl ester, the volatiles were removed under reduced pressure. The residue was taken up in water and extracted with DCM (2 × 20 mL). The combined organic layers were dried over anhydrous Na_2_SO_4_ and concentrated to afford IN-4 as a colorless oil (885 mg, 75% yield). ^1^H NMR (400 MHz, CDCl_3_) δ 5.39–5.31 (m, 4H), 5.29–5.20 (m, 1H), 4.33–4.26 (m, 2H), 4.18–4.11 (m, 2H), 2.41–2.28 (m, 7H), 2.20 (dd, *J* = 12.0, 7.6 Hz, 1H), 2.09–1.93 (m, 9H), 1.79–1.68 (m, 1H), 1.67–1.53 (m, 6H), 1.40–1.20 (m, 40H), 1.00 (d, *J* = 6.8 Hz, 3H), 0.88 (t, *J* = 6.8 Hz, 6H).

#### 2.2.2. Preparation of Curcumin Prodrug (CUR-PRO)

Synthesis of CUR-PRO: Compound IN-4 (300 mg, 0.393 mmol) was dissolved in anhydrous DCM (10 mL). Curcumin (217 mg, 0.591 mmol), N, N-diisopropylethylamine (DIPEA, 102 mg, 0.786 mmol), and DMAP (48 mg, 0.393 mmol) were added at room temperature, and the mixture was cooled to 0 °C in an ice-water bath. A solution of EDC·HCl (113 mg, 0.591 mmol) in a minimal volume of DCM was added dropwise. After completion of the addition, the reaction mixture was allowed to warm gradually to 25 °C and stirred for 16 h. The reaction progress was monitored by TLC (petroleum ether/ethyl acetate = 3:1), which indicated complete consumption of the starting material (Rf = 0.3). The reaction was quenched with 1 N aqueous HCl (5 mL) and water (10 mL), and the mixture was extracted with DCM (2 × 10 mL). The combined organic layers were washed with saturated brine (10 mL), dried over anhydrous Na_2_SO_4_, filtered, and concentrated under reduced pressure. The crude residue was purified by flash column chromatography on silica gel (gradient elution: 25–30% ethyl acetate in petroleum ether) to afford CUR-PRO as a yellow oil (198 mg, 45.3% yield).

#### 2.2.3. Synthesis of Betulinic Acid Prodrug (BA-PRO)

Synthesis of allyl betulinate (BA-PRO-1): A mixture of betulinic acid (1.0 g, 2.19 mmol) and potassium carbonate (605 mg, 4.38 mmol, 2.0 equiv) in anhydrous N, N-dimethylformamide (DMF, 10 mL) was stirred at room temperature for 30 min under nitrogen. Allyl bromide (397 mg, 3.28 mmol, 1.5 equiv) was added dropwise, and the reaction mixture was stirred at 20 °C for 4 h and monitored by TLC (petroleum ether/ethyl acetate = 10:1, Rf of betulinic acid = 0.2). Upon completion, the mixture was poured into ice water (30 mL) with vigorous stirring. The precipitated solid was collected by filtration, washed with water (5 mL), and dried under vacuum to afford BA-PRO-1 as a pale yellow solid (1.03 g, 95.3%). ^1^H NMR (400 MHz, DMSO-d_6_) δ 5.99–5.84 (m, 1H), 5.75 (s, 1H), 5.36–5.26 (m, 1H), 5.27–5.18 (m, 1H), 4.69 (d, *J* = 2.4 Hz, 1H), 4.62–4.48 (m, 3H), 4.26 (d, *J* = 4.8 Hz, 1H), 2.98–2.91 (m, 1H), 2.21–2.11 (m, 2H), 1.85–1.72 (m, 2H), 1.65 (s, 3H), 1.62–1.22 (m, 15H), 1.17–1.06 (m, 2H), 1.03–0.96 (m, 1H), 0.93 (s, 3H), 0.89–0.80 (m, 7H), 0.76 (s, 3H), 0.67–0.60 (m, 4H).

Synthesis of carbonate intermediate BA-PRO-2: To a solution of BA-PRO-1 (500 mg, 1.00 mmol) in anhydrous DCM (10 mL) was added pyridine (796 mg, 10.06 mmol, 10.0 equiv). After cooling to 0 °C, a solution of 1-chloroethyl chloroformate (575 mg, 4.03 mmol, 4.0 equiv) in DCM (2 mL) was added dropwise. The mixture was allowed to warm to 20 °C and stirred for 1 h and monitored by TLC (petroleum ether/ethyl acetate = 15:1). The reaction was quenched with 1 N aqueous HCl (10 mL) and water (10 mL), and the mixture was extracted with DCM (2 × 20 mL). The combined organic layers were washed with saturated brine (20 mL), dried over Na_2_SO_4_, and concentrated to give crude BA-PRO-2 as a pale yellow solid (585 mg, 96.5%), which was used directly in the next step.

Synthesis of coupled ester BA-PRO-3: A mixture of BA-PRO-2 (200 mg, 0.331 mmol), IN-4 (253 mg, 0.331 mmol, 1.0 equiv), cesium carbonate (Cs_2_CO_3_, 108 mg, 0.331 mmol, 1.0 equiv), and tetrabutylammonium iodide (TBAI, 122 mg, 0.331 mmol, 1.0 equiv) in anhydrous toluene (10 mL) was heated at 90 °C under nitrogen for 3 h and monitored by TLC (petroleum ether/ethyl acetate = 10:1). After cooling to ambient temperature, the mixture was diluted with water (20 mL) and extracted with ethyl acetate (2 × 20 mL). The combined organic extracts were washed with saturated brine (20 mL), dried over Na_2_SO_4_, and concentrated. Purification by flash column chromatography (petroleum ether/ethyl acetate = 10:1) afforded BA-PRO-3 as a colorless oil (338 mg, 77.0%).

Deprotection to afford BA-PRO: A solution of BA-PRO-3 (338 mg, 0.254 mmol) in degassed DCM/ethyl acetate (1:1, *v*/*v*, 10 mL) was treated with tetrakis(triphenylphosphine)palladium (0) [Pd(PPh_3_)_4_, 59 mg, 0.050 mmol, 0.2 equiv] and 1,3-dimethylbarbituric acid (79 mg, 0.508 mmol, 2.0 equiv) under nitrogen. The mixture was stirred at 25 °C for 2 h and monitored by TLC (petroleum ether/ethyl acetate = 3:1). After completion, the solvents were removed under reduced pressure. The crude residue was purified by flash column chromatography (15% ethyl acetate in petroleum ether) to yield BA-PRO as a colorless oil (249 mg, 76.0%).

#### 2.2.4. Preparation of Oleanolic Acid Prodrug (OA-PRO)

Synthesis of allyl oleanolate (OA-PRO-1): A mixture of oleanolic acid (1.0 g, 2.19 mmol) and potassium carbonate (605 mg, 4.38 mmol, 2.0 equiv) in anhydrous DMF (10 mL) was stirred at room temperature for 30 min under nitrogen. Allyl bromide (397 mg, 3.28 mmol, 1.5 equiv) was added dropwise, and the reaction mixture was stirred at 20 °C for 4 h and monitored by TLC (petroleum ether/ethyl acetate = 10:1, Rf of oleanolic acid = 0.2). Upon completion, the mixture was poured into ice water (30 mL) with vigorous stirring. The precipitated solid was collected by vacuum filtration, washed with water (5 mL), and dried in vacuo to afford OA-PRO-1 as a pale yellow solid (1.01 g, 93.4%). ^1^H NMR (400 MHz, DMSO-d_6_) δ 5.97–5.79 (m, 1H), 5.36–5.24 (m, 1H), 5.22–5.17 (m, 2H), 4.56–4.42 (m, 2H), 4.28 (d, *J* = 5.2 Hz, 1H), 3.05–2.95 (m, 1H), 2.86–2.75 (m, 1H), 2.04–1.92 (m, 1H), 1.85–1.77 (m, 2H), 1.69–1.12 (m, 15H), 1.10 (s, 3H), 1.08–0.98 (m, 2H), 0.95–0.78 (m, 13H), 0.73–0.60 (m, 7H).

Synthesis of carbonate intermediate OA-PRO-2: To a solution of OA-PRO-1 (200 mg, 0.40 mmol) in anhydrous DCM (10 mL) was added pyridine (318 mg, 4.02 mmol, 10.0 equiv). The reaction mixture was cooled to 0 °C in an ice bath, and a solution of 1-chloroethyl chloroformate (230 mg, 1.61 mmol, 4.0 equiv) in DCM (1 mL) was added dropwise over 5 min. The reaction was allowed to warm gradually to 20 °C and stirred for 1 h and monitored by TLC (petroleum ether/ethyl acetate = 15:1). The reaction was quenched by the sequential addition of 1 N aqueous HCl (10 mL) and water (10 mL). The mixture was extracted with DCM (2 × 20 mL), and the combined organic extracts were washed with saturated brine (20 mL), dried over anhydrous Na_2_SO_4_, filtered, and concentrated under reduced pressure to give crude OA-PRO-2 as a pale yellow solid (242 mg, quantitative yield), which was used directly in the next step. ^1^H NMR (400 MHz, CDCl_3_) δ 6.46–6.39 (m, 1H), 5.98–5.82 (m, 1H), 5.36–5.17 (m, 3H), 4.61–4.35 (m, 3H), 2.96–2.82 (m, 1H), 2.03–1.93 (m, 1H), 1.90–1.82 (m, 5H), 1.78–1.52 (m, 10H), 1.48–1.17 (m, 6H), 1.14 (s, 3H), 1.09–0.80 (m, 18H), 0.73 (s, 3H).

Synthesis of coupled ester OA-PRO-3: A mixture of OA-PRO-2 (211 mg, 0.349 mmol), IN-4 (294 mg, 0.349 mmol, 1.0 equiv), Cs_2_CO_3_ (114 mg, 0.349 mmol, 1.0 equiv), and TBAI (129 mg, 0.349 mmol, 1.0 equiv) in anhydrous toluene (10 mL) was heated at 90 °C and stirred vigorously under nitrogen for 3 h and monitored by TLC (petroleum ether/ethyl acetate = 10:1). Upon complete consumption of OA-PRO-2, the mixture was cooled to ambient temperature and diluted with water (20 mL). The mixture was extracted with ethyl acetate (2 × 20 mL), and the combined organic layers were washed with saturated brine (20 mL), dried over anhydrous Na_2_SO_4_, filtered, and concentrated. The crude residue was purified by flash column chromatography on silica gel (petroleum ether/ethyl acetate = 10:1) to afford OA-PRO-3 as a colorless oil (309 mg, 66.5% yield).

Deprotection to afford OA-PRO: A solution of OA-PRO-3 (309 mg, 0.232 mmol) in degassed DCM/ethyl acetate (1:1, *v*/*v*, 10 mL) was treated with Pd(PPh_3_)_4_ (54 mg, 0.046 mmol, 0.2 equiv) and 1,3-dimethylbarbituric acid (73 mg, 0.465 mmol, 2.0 equiv) under nitrogen. The resulting mixture was stirred at 25 °C for 2 h and monitored by TLC (petroleum ether/ethyl acetate = 3:1). After complete deprotection, the volatiles were removed under reduced pressure. The crude product was purified by flash column chromatography on silica gel (15% ethyl acetate in petroleum ether) to furnish OA-PRO as a colorless, viscous oil (180 mg, 60.0% yield).

### 2.3. Comparative Pharmacokinetic Studies of Prodrugs in Rats

#### 2.3.1. Animals and Ethics

All animal experiments were approved by the Institutional Animal Care and Use Committee (IACUC) of the Suzhou Institute of Materia Medica (Approval Nos.: 2023-10-HJ-13, approved on 16 October 2023; and 2025-02-HJ-14, approved on 10 February 2025). Male Sprague–Dawley rats were obtained from Shanghai Jihui Laboratory Animal Care Co., Ltd. (Shanghai, China) Rats aged 7–8 weeks and weighing 180–250 g were used for pharmacokinetic studies, whereas rats aged 8–12 weeks and weighing 300–400 g were used for lymphatic transport studies. Animals were housed under standard laboratory conditions (22 ± 2 °C, 55 ± 10% relative humidity, 12 h light/dark cycle) with free access to water. Animals used for pharmacokinetic studies were fasted for 16 h before oral dosing, and food was returned 4 h after dosing; animals used for lymphatic transport studies were not fasted. The experimental unit was an individual rat, and the sample size was set at *n* = 3 per group. This study was designed as an exploratory proof-of-concept investigation, and the pharmacokinetic data obtained were primarily used to provide a preliminary assessment of the feasibility of the prodrug strategy for improving oral exposure and apparent bioavailability. More comprehensive pharmacokinetic evaluation would be warranted in subsequent developability studies.

#### 2.3.2. Study Design for Curcumin Prodrug (CUR-PRO)

This study compared the oral pharmacokinetic behavior of the curcumin prodrug (CUR-PRO) with that of its parent drug, curcumin, after oral administration in male Sprague–Dawley rats. To address the potential influence of vehicle composition on the oral absorption of lipophilic compounds, CUR-PRO and curcumin were evaluated under three paired vehicle conditions. CUR-PRO was administered by oral gavage at 40 mg/kg, corresponding to 13.24 mg/kg curcumin equivalents, whereas free curcumin was administered orally at 40 mg/kg. The same dose volume (10 mL/kg) and sampling schedule were used for all oral groups. An intravenous curcumin group (4 mg/kg, 1 mL/kg) was included as a reference for estimating the apparent oral bioavailability of released free curcumin exposure (*n* = 3 per group).

Three oral vehicles were evaluated under matched vehicle conditions for CUR-PRO and curcumin: Vehicle 1, PEG 400/Transcutol HP/Solutol HS 15/0.9% saline (30:10:7:53, *v*/*v*/*v*/*v*); Vehicle 2, oleic acid/Tween 80/0.9% saline (2:1.25:96.75, *v*/*v*/*v*); and Vehicle 3, 0.5% CMC-Na. Vehicle 1 produced clear preparations for both CUR-PRO and curcumin. Vehicle 2 produced emulsion/suspension preparations for both compounds. In Vehicle 3, CUR-PRO and curcumin were both formulated as suspensions. These vehicles were selected to compare CUR-PRO and curcumin under paired vehicle conditions while also examining whether the observed exposure enhancement was preserved across distinct vehicle environments.

The intravenous curcumin formulation was prepared by sequential dissolution of free curcumin in a mixture of DMSO, ethanol, propylene glycol, PEG 400, and 0.9% saline (0.5:20:10:40:30, *v*/*v*/*v*/*v*/*v*), followed by filtration through a 0.22 μm membrane.

Blood samples (0.2 mL) were collected from the jugular vein at 0.25, 0.5, 1, 1.5, 2.5, 4, 6, 8, and 24 h after oral dosing. For the intravenous curcumin reference group, blood samples were collected at 0.083, 0.25, 0.5, 1, 2, 4, 8, and 24 h after dosing.

Plasma concentrations of free curcumin and intact CUR-PRO were separately quantified by LC-MS/MS as described below. For curcumin-dosed groups, free curcumin was quantified. For CUR-PRO-dosed groups, both released free curcumin and intact CUR-PRO were independently quantified at each time point. The final pharmacokinetic comparison for curcumin exposure was based on free curcumin concentrations, whereas intact CUR-PRO exposure was analyzed separately to evaluate vehicle-dependent prodrug absorption.

Bioanalysis: Plasma concentrations of free curcumin and intact CUR-PRO were quantified by LC-MS/MS. Briefly, 20 μL of plasma was vortex-mixed with 200 μL of acetonitrile for 5 min and then centrifuged (12,000 rpm, 5 min, 4 °C). The supernatant was transferred to a 96-well plate for injection and analysis.

Quantification was performed using an HPLC system (LC-30AD, Shimadzu, Kyoto, Japan) coupled to a triple quadrupole mass spectrometer (QTRAP 5500, AB Sciex, Framingham, MA, USA).

For curcumin, chromatographic separation was achieved on a Luna Omega 3 Polar C18 column (Phenomenex, Torrance, CA, USA) (2.1 × 100 mm, 100 Å, 3 μm) using a gradient mobile phase composed of mobile phase A: 2 mmol/L ammonium formate in 0.05% formic acid (*v*/*v*) and mobile phase B: 0.1% formic acid in acetonitrile, at a flow rate of 0.4 mL/min and a column temperature of 40 °C. The gradient elution profile for curcumin was as follows: solvent B was held at 35% at 0 min, increased linearly to 95% by 1.2 min, maintained at 95% until 2.5 min, returned to 35% at 2.6 min, and held constant until 3.0 min. Detection was carried out by multiple reaction monitoring (MRM) in positive electrospray ionization (ESI+) mode. The MRM transition for curcumin was *m*/*z* 369.2 → 245.0, with a collision energy (CE) of 16 V and a declustering potential (DP) of 100 V. Dexamethasone was used as the internal standard (IS), and the MRM transition for the IS was *m*/*z* 393.3 → 147.1, with a CE of 30 V and a DP of 10 V. Quantification was performed using the peak-area ratio of curcumin to the IS. The analytical method validation for curcumin is described in Supporting Information S1.

For intact CUR-PRO, chromatographic separation was performed on a Kinetex HILIC column (Phenomenex, Torrance, CA, USA) (3.0 × 100 mm, 2.6 μm) using a gradient mobile phase composed of mobile phase A (10 mmol/L ammonium formate in water) and mobile phase B (0.1% formic acid in acetonitrile), at a flow rate of 0.6 mL/min and a column temperature of 40 °C. The gradient elution profile was as follows: solvent B was held at 95% from 0 to 0.5 min, decreased linearly to 60% by 0.6 min, maintained at 60% until 1.4 min, returned to 95% at 1.5 min, and held at 95% until 2.0 min. Detection was performed in negative electrospray ionization (ESI−) mode using MRM of the transition *m*/*z* 1111.7 → 961.6, with a collision energy (CE) of −45 V and a declustering potential (DP) of −20 V.

#### 2.3.3. Study Design for Betulinic Acid Prodrug (BA-PRO)

This study compared betulinic acid exposure after oral administration of the betulinic acid prodrug (BA-PRO) or its parent drug, betulinic acid (*n* = 3 per group).

Group 1 (BA-PRO PO): Received an oral gavage of BA-PRO in blank vehicle at 40 mg/kg (10 mL/kg, equivalent to 14.2 mg/kg betulinic acid).

Group 2 (Betulinic Acid PO): Received an oral gavage of free betulinic acid in blank vehicle at 40 mg/kg (10 mL/kg).

Vehicle: The oral vehicle consisted of rice bran oil, Transcutol^®^ HP, Solutol^®^ HS 15, and Lipoid^®^ E 80 at a ratio of 44:11:0.55:2.2 (*v*/*v*/*v*/*w*). This lipid-rich vehicle was selected to accommodate the high lipophilicity of BA-PRO and betulinic acid and to ensure complete dissolution.

Sampling: Blood samples (0.2 mL) were collected at 0.5, 1, 1.5, 2, 4, 6, 8, and 24 h post-dose.

Bioanalysis: Plasma concentrations of betulinic acid were quantified by LC-MS/MS. Briefly, 20 μL of plasma was vortex-mixed with 200 μL of methanol for 5 min, followed by centrifugation at 12,000 rpm for 5 min at 4 °C. The resulting supernatant was transferred to a clean 96-well plate for injection and analysis.

Quantification was performed using an HPLC system (LC-30AD, Shimadzu, Kyoto, Japan) coupled to a triple quadrupole mass spectrometer (QTRAP 5500, AB Sciex, Framingham, MA, USA).

For betulinic acid, chromatographic separation was achieved on an InfinityLab Poroshell 120 EC-C18 column (Agilent Technologies, Santa Clara, CA, USA) (2.1 × 100 mm, 1.9 μm) using an isocratic mobile phase composed of 5% mobile phase A (0.1% formic acid in water) and 95% mobile phase B (0.1% formic acid in acetonitrile), at a flow rate of 0.4 mL/min and a column temperature of 40 °C. Detection was performed in negative electrospray ionization (ESI−) mode using multiple reaction monitoring (MRM) of the transition *m*/*z* 455.4 → 455.4, with a collision energy (CE) of −20 V and a declustering potential (DP) of −200 V.

For intact BA-PRO, reliable LC-MS/MS quantification could not be achieved under the tested analytical conditions, despite optimization of ionization mode, mobile-phase composition, and chromatographic parameters. Therefore, BA-PRO pharmacokinetics were assessed based on plasma concentrations of the corresponding parent drug, betulinic acid.

#### 2.3.4. Study Design for Oleanolic Acid Prodrug (OA-PRO)

This study compared oleanolic acid exposure after oral administration of the oleanolic acid prodrug (OA-PRO) or its parent drug, oleanolic acid, in male Sprague–Dawley rats (*n* = 3 per group).

Group 1 (OA-PRO PO): Received an oral gavage of OA-PRO solution at 40 mg/kg (10 mL/kg, equivalent to 14.2 mg/kg oleanolic acid).

Group 2 (Oleanolic Acid PO): Received an oral gavage of free oleanolic acid solution at 40 mg/kg (10 mL/kg).

Vehicle: The oral vehicle consisted of oleic acid, olive oil, Transcutol^®^ HP, and Solutol^®^ HS 15 at a volume ratio of 1:40:10:1.5 (*v*/*v*/*v*/*v*). OA-PRO and oleanolic acid were prepared in the same oral vehicle for oral administration.

Sampling: Blood samples (0.2 mL) were collected at 0.5, 1, 1.5, 2, 4, 6, 8, and 24 h post-dose.

Bioanalysis: Plasma concentrations of oleanolic acid were quantified by LC-MS/MS. Briefly, 20 μL of plasma was vortex-mixed with 200 μL of methanol for 5 min, followed by centrifugation at 12,000 rpm for 5 min at 4 °C. The resulting supernatant was transferred to a clean 96-well plate for injection and analysis.

Quantification was performed using an HPLC system (LC-30AD, Shimadzu, Kyoto, Japan) coupled to a triple quadrupole mass spectrometer (QTRAP 5500, AB Sciex, Framingham, MA, USA).

For oleanolic acid, chromatographic separation was achieved on an InfinityLab Poroshell 120 EC-C18 column (Agilent Technologies, Santa Clara, CA, USA) (2.1 × 100 mm, 1.9 μm) using a gradient elution profile: the mobile phase began at 90% A (0.1% formic acid in water) and 10% B (0.1% formic acid in acetonitrile), was linearly shifted to 5% A and 95% B over 0.4 min, held at this composition for 1.2 min, rapidly returned to the initial conditions over 0.1 min, and equilibrated for 0.3 min before the next injection. The flow rate was maintained at 0.4 mL/min, and the column temperature was controlled at 40 °C. Detection was performed in negative electrospray ionization (ESI−) mode using multiple reaction monitoring (MRM) of the transition *m*/*z* 455.4 → 407.2, with a collision energy (CE) of −55 V and a declustering potential (DP) of −130 V.

Similarly, intact OA-PRO could not be reliably quantified by LC-MS/MS under the tested analytical conditions, despite optimization of ionization mode, mobile-phase additives, and chromatographic parameters. Therefore, OA-PRO pharmacokinetics were assessed based on plasma concentrations of the corresponding parent drug, oleanolic acid.

#### 2.3.5. Pharmacokinetic and Statistical Analysis

For all pharmacokinetic studies, plasma was separated by centrifugation (8000 rpm, 5 min, 4 °C) and stored at −80 °C until analysis. Pharmacokinetic parameters, including the maximum plasma concentration (C_max_), time to reach C_max_ (T_max_), area under the plasma concentration-time curve (AUC_0-last_ and AUC_0-∞_), terminal half-life (t_1/2_), mean residence time (MRT_inf_), and apparent oral bioavailability (F, when applicable), were calculated by noncompartmental analysis using Phoenix WinNonlin(version 8.1, Pharsight, Mountain View, CA, USA). Data are presented primarily as descriptive statistics (mean ± SD). Given the exploratory proof-of-concept nature of the study and the small sample size, emphasis was placed on the magnitude and consistency of observed exposure trends rather than formal hypothesis testing.

For the curcumin-related pharmacokinetic analysis, free curcumin concentrations were used to calculate pharmacokinetic parameters for both curcumin-dosed and CUR-PRO-dosed groups. In CUR-PRO-dosed groups, intact CUR-PRO concentrations were analyzed separately, and intact CUR-PRO exposure was not combined with free curcumin exposure in the final pharmacokinetic comparison. Apparent oral bioavailability of released free curcumin exposure was estimated using the intravenous curcumin reference group and dose normalization based on curcumin or curcumin-equivalent dose.

For BA-PRO- and OA-PRO-dosed groups, pharmacokinetic parameters were calculated from plasma concentrations of the corresponding released parent drugs, betulinic acid and oleanolic acid, respectively, because reliable quantification of intact BA-PRO and OA-PRO in plasma could not be established under the current analytical conditions. Fold increases in exposure were calculated from dose-normalized AUC_0-last_ values using the parent-drug-equivalent dose of each prodrug and the dose of the corresponding parent drug.

### 2.4. Investigation of Intestinal Lymphatic Transport

#### 2.4.1. Study Design and Surgical Procedure

To provide direct evidence supporting the lymphatic transport mechanism, intestinal lymphatic transport of CUR-PRO was evaluated in non-fasted male Sprague–Dawley rats (300–400 g, *n* = 3 per group) using a mesenteric lymph duct cannulation model. Four oral groups were included under two matched vehicle conditions: CUR-PRO in Vehicle 1, curcumin in Vehicle 1, CUR-PRO in Vehicle 2, and curcumin in Vehicle 2. CUR-PRO was administered by oral gavage at 80 mg/kg, corresponding to 26.5 mg/kg curcumin equivalents, whereas free curcumin was administered at 30 mg/kg. The dosing volume was 10 mL/kg for all groups. Actual curcumin-equivalent doses were calculated based on individual body weights and used for dose-normalized lymphatic recovery calculations.

Vehicle 1 consisted of PEG 400/Transcutol HP/Solutol HS 15/0.9% saline (30:10:7:53, *v*/*v*/*v*/*v*) and produced clear dosing preparations for both CUR-PRO and curcumin. Vehicle 2 consisted of oleic acid/Tween 80/0.9% saline (2:1.25:96.75, *v*/*v*/*v*) and produced homogeneous emulsion-type dosing preparations. After a 15 min post-dosing absorption period, rats were anesthetized for lymph duct cannulation. A midline abdominal incision was made to expose the mesenteric lymph duct adjacent to the superior mesenteric artery. Under a stereomicroscope, the lymph duct was isolated and cannulated with a polyethylene catheter. The catheter was exteriorized, and the abdominal contents were returned to their original position.

#### 2.4.2. Lymph Collection and Bioanalysis

Mesenteric lymph was collected continuously at 1 h intervals for 6 h after dosing (0–1, 1–2, 2–3, 3–4, 4–5, and 5–6 h). The volume of lymph collected during each interval was recorded and used to calculate the amount of analyte recovered in lymph.

For bioanalysis, a 40 μL aliquot of each lymph sample was mixed with 460 μL of acetonitrile. The mixture was vortexed thoroughly and centrifuged at 12,000 rpm for 5 min. A 200 μL portion of the supernatant was transferred to a clean vial for HPLC-UV analysis.

Intact CUR-PRO and free curcumin in lymph samples were quantified by HPLC-UV using reference standards. Chromatographic separation was achieved on a YMC-Pack ODS-AQ column (YMC Co., Ltd., Kyoto, Japan) (4.6 × 150 mm, 5 μm, 12 nm) maintained at 35 °C. The mobile phase consisted of methanol (B) and 0.1% aqueous formic acid (D), delivered at 1.0 mL/min with the following gradient program: 0–5 min, 65% B to 97% B; 5–6 min, 97% B to 100% B; 6–30 min, 100% B; and 31–38 min, re-equilibration at 65% B. The injection volume was 10 μL, and detection was performed using a photodiode array detector at 412 nm. Representative lymph sample photographs and representative HPLC-UV chromatograms are provided in the Supporting Information (Figures S1 and S2).

In addition to the reference-standard-confirmed curcumin and CUR-PRO peaks, all additional UV-absorbing peaks detected at 412 nm between approximately 3.0 and 30.0 min were integrated after excluding the confirmed curcumin and CUR-PRO peaks. These signals were reported as unassigned 412 nm UV-absorbing peaks and were semi-quantitatively expressed as curcumin-equivalent amounts using the curcumin calibration response. Because no confirmatory MS/MS evidence was obtained for these minor peaks, they were not assigned to specific metabolites or degradation products.

#### 2.4.3. Lymphatic Recovery Calculations

For each collection interval, the recovered amount was calculated as the analyte concentration multiplied by the corresponding lymph volume. Intact CUR-PRO was converted to curcumin equivalents using the molecular-weight ratio of curcumin to CUR-PRO. Total curcumin-equivalent lymphatic recovery was calculated as the sum of intact CUR-PRO expressed as curcumin equivalents, free curcumin, and unassigned 412 nm UV-absorbing peaks expressed as curcumin equivalents. The percentage of the administered dose recovered in lymph was calculated using the actual curcumin-equivalent dose for each animal. Data are presented as mean ± SD (*n* = 3).

### 2.5. Stability and Conversion Studies of CUR-PRO in Biorelevant and Biological Media

To further characterize the stability and conversion behavior of CUR-PRO, complementary in vitro assays were conducted in simulated gastrointestinal media, rat liver microsomes, and rat plasma. These experiments were designed to evaluate the chemical stability of CUR-PRO and curcumin under enzyme-free gastric and intestinal conditions, their susceptibility to pancreatic lipase-mediated digestion in FaSSIF, their metabolic stability in rat liver microsomes, and their plasma stability and LPL-mediated conversion profiles.

#### 2.5.1. Experimental Conditions for Stabilityin Simulated Gastrointestinal Media and Lipase-Containing FaSSIF

CUR-PRO and curcumin were separately incubated in fed-state simulated intestinal fluid (FeSSIF, pH 5.0), fasted-state simulated intestinal fluid (FaSSIF, pH 6.5), and fasted-state simulated gastric fluid (FaSSGF, pH 1.6) at 37 °C. To evaluate lipase-mediated intestinal digestion, additional incubations were performed in FaSSIF supplemented with porcine pancreatic lipase. Briefly, each test compound was added to pre-equilibrated medium, and incubations were performed in triplicate. Samples were collected at 0, 5, 10, 15, 30, 60, 90, 120, and 180 min. At each time point, aliquots were immediately quenched with acetonitrile, vortex-mixed, centrifuged, and analyzed by LC–MS/MS. The remaining percentage of CUR-PRO or curcumin was calculated relative to the corresponding 0 min sample. For CUR-PRO incubations, released curcumin was also monitored. In the lipase-containing FaSSIF system, formation of a curcumin monoglyceride-like derivative was observed; because an authentic standard was not available, this species was monitored semi-quantitatively using the curcumin calibration curve and is described qualitatively.

#### 2.5.2. Experimental Conditions for Rat Liver Microsomal Stability

CUR-PRO and curcumin were incubated with pooled rat liver microsomes (0.25 mg protein/mL) in 100 mM phosphate buffer at 37 °C in the presence of NADPH (1 mM). The final substrate concentration was 10 μM, and verapamil was used as a positive control. Aliquots were collected at 0.5, 5, 15, 30, 45, and 60 min and quenched with cold acetonitrile. After centrifugation, the supernatants were analyzed by LC-MS/MS, and the percentage remaining was calculated relative to the 0.5 min sample.

#### 2.5.3. Experimental Conditions for Rat Plasma Stability and LPL-Enhanced Conversion

The plasma stability of CUR-PRO and curcumin was evaluated in rat plasma at 37 °C and 4 °C. CUR-PRO or curcumin was spiked into rat plasma and incubated in triplicate. Samples were collected at 0, 5, 15, 30, 60, 90, 120, and 180 min. To evaluate LPL-mediated conversion, parallel incubations were performed in rat plasma supplemented with LPL at 37 °C. Briefly, the plasma incubation mixture was prewarmed for 5 min, followed by addition of LPL solution (10,000 U/mL) and continued incubation at 37 °C. At each time point, aliquots were withdrawn and immediately quenched with acetonitrile to precipitate plasma proteins. After vortex-mixing and centrifugation, the supernatants were analyzed by LC–MS/MS. For CUR-PRO incubation samples, both intact CUR-PRO and released curcumin were monitored. The remaining percentage of CUR-PRO or curcumin was calculated relative to the 0 min concentration.

## 3. Results and Discussion

### 3.1. Synthesis of the Lipid-Based Promoiety (IN-4)

**Scheme 1:**
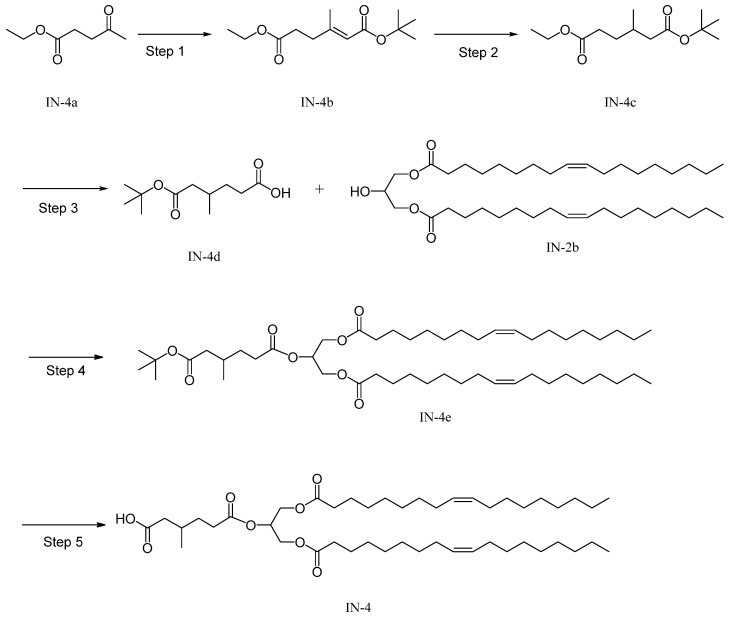
Synthesis of the lipid-based promoiety (IN-4).

The common lipid-based promoiety IN-4, designed to increase lipophilicity and to mimic a triacylglycerol-processing substrate, was synthesized in five steps from commercially available ethyl levulinate (IN-4a), as shown in Scheme 1. A Horner–Wadsworth–Emmons olefination between tert-butyl (dimethoxyphosphoryl)acetate and IN-4a produced the α, β-unsaturated ester IN-4b in 77.3% yield. Catalytic hydrogenation then afforded the saturated diester IN-4c, and selective hydrolysis followed by acidification gave mono-acid mono-tert-butyl ester IN-4d in 89.6% yield over two steps. Installation of the 1,3-diolein-derived lipid fragment by Steglich esterification yielded protected intermediate IN-4e in 50.6% yield, and final deprotection furnished IN-4 in 75% yield. Overall, this route provided a practical convergent entry to the lipid promoiety used throughout the study. The chemical characterization data for IN-4 are provided in the Supporting Information (Figure S3).

### 3.2. Synthesis of Prodrugs via a Convergent Coupling Strategy

**Scheme 2:**
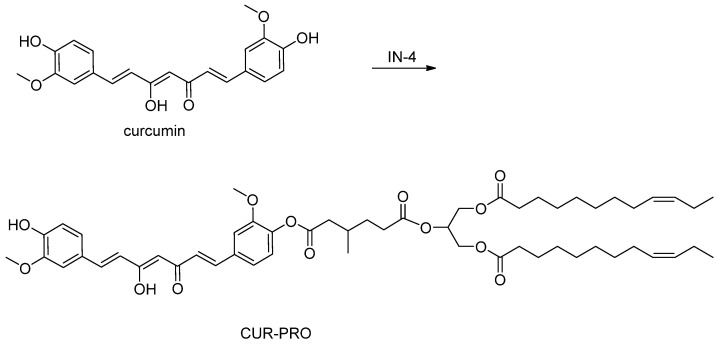
Synthesis of CUR-PRO.

**Scheme 3:**
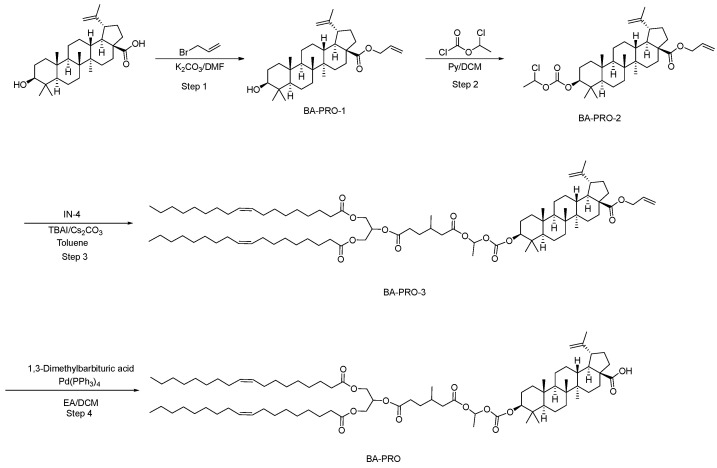
Synthesis of BA-PRO.

**Scheme 4:**
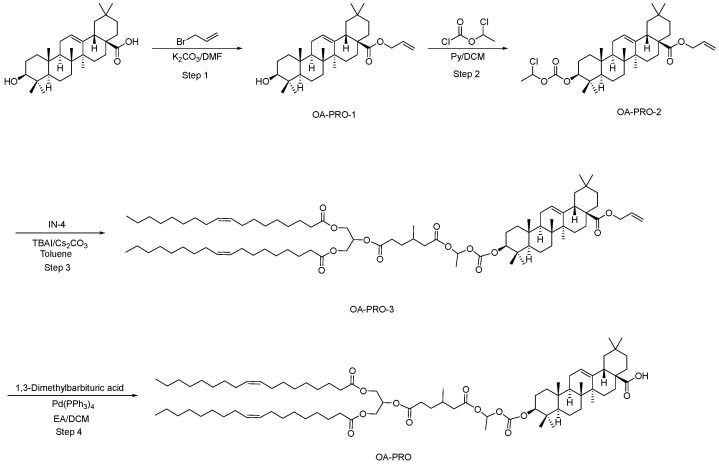
Synthesis of OA-PRO.

Using lipid carboxylic acid IN-4 as a common promoiety, three conjugates—CUR-PRO, BA-PRO, and OA-PRO—were synthesized through convergent coupling routes (Scheme 2, Scheme 3 and Scheme 4). This modular design enabled direct comparison of a shared lipid scaffold across three selected natural products spanning a polyphenol and two pentacyclic triterpenoids. Because the present manuscript focuses primarily on proof-of-concept synthesis and exploratory pharmacokinetic evaluation, the structural characterization reported here should be interpreted in that context.

#### 3.2.1. Synthesis and Characterization of Curcumin Prodrug (CUR-PRO)

The hydroxyl group of curcumin was directly conjugated to the carboxylic acid of IN-4 under classic Steglich esterification conditions. A mixture of IN-4, curcumin, N,N-diisopropylethylamine (DIPEA), and 4-dimethylaminopyridine (DMAP) in dichloromethane was activated with 1-(3-dimethylaminopropyl)-3-ethylcarbodiimide hydrochloride (EDC·HCl) at 0 °C and then allowed to react at room temperature. This one-step coupling afforded the target prodrug CUR-PRO as a yellow oil in 45.3% yield after purification by flash chromatography. CUR-PRO was characterized by ^1^H NMR, ^13^C NMR, and time-of-flight mass spectrometry (TOF-MS). Its purity was determined by HPLC-UV analysis. *m*/*z* [M − H]^−^ = 1111.7510. HPLC purity: 93.2%. The chemical characterization data for CUR-PRO are provided in the Supporting Information (Figure S4).

CUR-PRO was characterized by NMR spectroscopy as follows. ^1^H NMR (400 MHz, CDCl_3_) δ 7.61 (dd, *J* = 16.0, 3.2 Hz, 2H, CH=CHCO), 7.19–7.09 (m, 3H, Ar-H), 7.08–7.01 (m, 2H, Ar-H), 6.94 (d, *J* = 8.0 Hz, 1H, Ar-H), 6.59–6.45 (m, 2H, CH=CHCO), 5.94–5.77 (m, 2H, OCH=CH), 5.41–5.23 (m, 5H, olefinic H and glycerol CH), 4.35–4.26 (m, 2H, glycerol CH_2_O), 4.20–4.11 (m, 2H, glycerol CH_2_O), 3.95 (s, 3H, OCH_3_), 3.87 (s, 3H, OCH_3_), 2.64–2.53 (m, 1H, CH of linker), 2.51–2.36 (m, 3H, CH_2_CO/CH of linker), 2.31 (t, *J* = 7.6 Hz, 4H, 2 × CH_2_COO), 2.05–1.94 (m, 8H, allylic CH_2_), 1.69–1.56 (m, 7H, aliphatic CH_2_ and CH_3_CH), 1.34–1.24 (m, 40H, long-chain methylene protons), 1.09 (d, *J* = 6.7 Hz, 3H, CHCH_3_), 0.88 (t, *J* = 6.8 Hz, 6H, 2 × CH_3_).^13^C NMR (101 MHz, CDCl_3_) δ 181.82 (β-diketone C=O), 173.28, 172.55 (ester C=O), 151.42, 148.04, 146.84 (oxygenated aromatic carbons), 141.20–101.50 (aromatic and olefinic carbons), 69.19, 62.05 (glycerol carbons), 55.96, 55.81 (methoxy carbons), 41.07, 34.01 (linker carbons), 31.90–14.11 (aliphatic carbons).

#### 3.2.2. Synthesis and Characterization of Betulinic Acid Prodrug (BA-PRO)

For the pentacyclic triterpenoid betulinic acid, a slightly modified sequence was employed to address its carboxyl group. First, the carboxylic acid was protected as its allyl ester (BA-PRO-1) by reaction with allyl bromide and K_2_CO_3_ in DMF, giving a 95.3% yield. Subsequently, the C-3 hydroxyl group was activated by conversion to a reactive mixed carbonate intermediate (BA-PRO-2) using 1-chloroethyl chloroformate and pyridine. This key intermediate was then coupled with IN-4 via nucleophilic substitution in toluene at 90 °C, catalyzed by Cs_2_CO_3_ and tetrabutylammonium iodide (TBAI), to yield the fully protected conjugate BA-PRO-3 in 77.0% yield. Finally, the allyl protecting group was cleanly removed under mild conditions using tetrakis(triphenylphosphine)palladium and 1,3-dimethylbarbituric acid, delivering the final betulinic acid prodrug BA-PRO as a colorless oil in 76.0% yield from BA-PRO-3. BA-PRO was characterized by ^1^H NMR and ^13^C NMR spectroscopy and time-of-flight mass spectrometry (TOF-MS), and its purity was determined by HPLC-UV analysis. *m*/*z* [M − H]^−^ = 1287.9970. HPLC purity: 96.2%. The chemical characterization data for BA-PRO are provided in the Supporting Information (Figure S5). BA-PRO was characterized by NMR spectroscopy as follows. ^1^H NMR (400 MHz, CDCl_3_) δ 6.82–6.70 (m, 1H, olefinic H), 5.41–5.20 (m, 5H, olefinic H and glycerol CH), 4.74 (s, 1H, exomethylene H), 4.61 (s, 1H, exomethylene H), 4.39–4.25 (m, 3H, glycerol CH_2_O and OCH), 4.18–4.09 (m, 2H, glycerol CH_2_O), 3.08–2.92 (m, 1H, triterpenoid CH), 2.41–1.91 (m, 22H, CH_2_COO, allylic CH_2_, and aliphatic CH), 1.73–1.23 (m, 71H, long-chain methylene and aliphatic protons), 1.00–0.81 (m, 24H, methyl protons and terminal CH_3_).^13^C NMR (101 MHz, CDCl_3_) δ 173.27, 172.47 (ester carbonyl carbons), 153.16, 150.34 (oxygenated quaternary carbons), 130.02, 129.72, 109.77 (olefinic carbons), 91.16, 86.03, 69.16, 62.04 (oxygenated carbons), 56.36–34.01 (triterpenoid skeleton and linker carbons), 32.15–14.11 (aliphatic carbons of the triterpenoid framework and oleoyl chains).

#### 3.2.3. Synthesis and Characterization of Oleanolic Acid Prodrug (OA-PRO)

A synthetic sequence analogous to that used for BA-PRO was applied to oleanolic acid, showing that the same synthetic sequence could be applied to a second pentacyclic triterpenoid acid. Oleanolic acid was similarly converted to its allyl ester (OA-PRO-1, 93.4% yield) and then to the corresponding chloroethyl carbonate intermediate (OA-PRO-2, quantitative yield). Coupling with IN-4 under the same conditions provided the intermediate conjugate OA-PRO-3 in 66.5% yield. Palladium-catalyzed deprotection of the allyl ester furnished the final oleanolic acid prodrug OA-PRO as a colorless oil in 60.0% yield. OA-PRO was characterized by ^1^H NMR and ^13^C NMR spectroscopy and time-of-flight mass spectrometry (TOF-MS), and its purity was determined by HPLC-UV analysis. *m*/*z* [M − H]^−^ = 1287.9996. HPLC purity: 96.3%. The chemical characterization data for OA-PRO are provided in the Supporting Information (Figure S6). OA-PRO was characterized by NMR spectroscopy as follows.

^1^H NMR (400 MHz, CDCl_3_) δ 6.83–6.70 (m, 1H, olefinic H), 5.42–5.20 (m, 6H, olefinic H and glycerol CH), 4.42–4.25 (m, 3H, glycerol CH_2_O and OCH), 4.20–4.08 (m, 2H, glycerol CH_2_O), 2.88–2.76 (m, 1H, triterpenoid CH), 2.38–2.27 (m, 7H, CH_2_COO and aliphatic CH), 2.23–2.10 (m, 1H, aliphatic CH), 2.03–1.98 (m, 8H, allylic CH_2_), 1.97–1.83 (m, 3H, aliphatic CH/CH_2_), 1.78–1.50 (m, 20H, aliphatic CH and CH_2_), 1.44–1.25 (m, 44H, long-chain methylene protons), 1.20–1.00 (m, 7H, aliphatic protons), 0.98–0.83 (m, 25H, methyl protons and terminal CH_3_), 0.75 (s, 3H, triterpenoid CH_3_). ^13^C NMR (101 MHz, CDCl_3_) δ 173.26, 172.48 (ester carbonyl carbons), 153.16 (carbonate-related quaternary carbon), 143.61, 130.02, 129.72, 122.49 (olefinic carbons), 91.18, 69.16, 62.03 (oxygenated carbons), 55.29–32.43 (triterpenoid skeleton and linker carbons), 31.91–14.12 (aliphatic carbons of the triterpenoid framework and oleoyl chains).

### 3.3. Pharmacokinetic Evaluation of Prodrugs

The pharmacokinetic profiles of the three synthesized prodrugs (CUR-PRO, BA-PRO, and OA-PRO) were evaluated after oral administration in rats and compared with those of their respective parent drugs. To address the concern that vehicle composition may influence oral absorption of lipophilic compounds, CUR-PRO was further examined under three matched vehicle conditions. Plasma exposure of released free curcumin and intact CUR-PRO was analyzed separately. The key pharmacokinetic parameters for free curcumin and intact CUR-PRO are summarized in Table 1 and Table 2, respectively. For BA-PRO and OA-PRO, intact prodrug concentrations could not be reliably quantified under the current analytical conditions; therefore, pharmacokinetic evaluation was based on plasma exposure of the released parent drugs, betulinic acid and oleanolic acid, respectively. The corresponding pharmacokinetic parameters are summarized in Table 3.

#### 3.3.1. Vehicle-Dependent Pharmacokinetics of Curcumin Prodrug (CUR-PRO)

**Figure 2:**
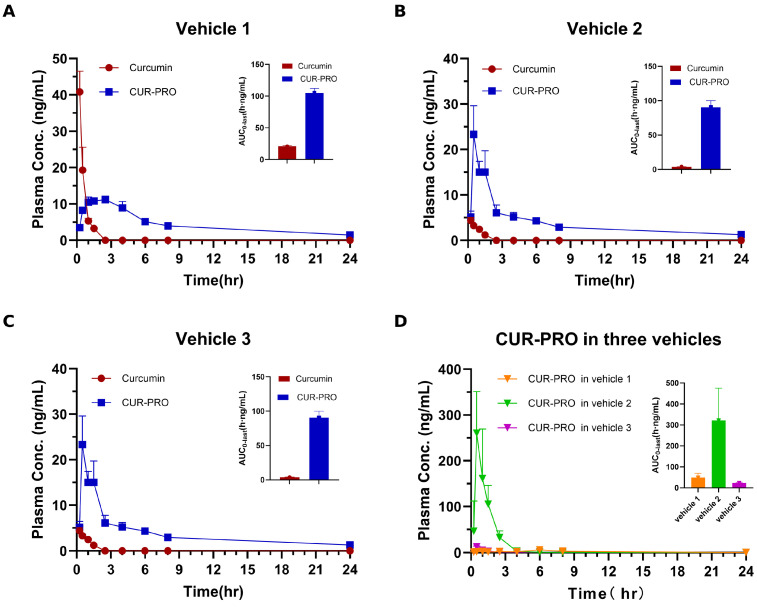
Plasma concentration–time profiles for free curcumin and intact CUR-PRO under matched vehicle conditions. (**A**–**C**) Free curcumin exposure after oral administration of CUR-PRO (40 mg/kg, equivalent to 13.2 mg/kg curcumin) or curcumin (40 mg/kg) in male Sprague–Dawley rats under Vehicle 1, Vehicle 2, and Vehicle 3, respectively. (**D**) Intact CUR-PRO exposure after oral administration of CUR-PRO under the three vehicle conditions. Data are presented as mean ± SD (*n* = 3).

CUR-PRO increased the systemic exposure of released free curcumin under all three matched vehicle conditions (Figure 2A–C; Table 1). In Vehicle 1, oral administration of CUR-PRO (40 mg/kg, equivalent to 13.24 mg/kg curcumin) produced an AUC_0-last_ of 105 ± 7.0 h·ng/mL for free curcumin, compared with 20.9 ± 1.3 h·ng/mL after administration of curcumin at 40 mg/kg, corresponding to a dose-normalized 15.0-fold increase. In Vehicle 2, the corresponding AUC_0-last_ values were 90.5 ± 9.5 and 3.86 ± 0.24 h·ng/mL, respectively, representing a 70.9-fold increase. In Vehicle 3, CUR-PRO yielded an AUC_0-last_ of 45.9 ± 1.8 h·ng/mL, compared with 2.56 ± 0.17 h·ng/mL for curcumin, corresponding to a 54.3-fold increase. The apparent oral bioavailability of released curcumin after CUR-PRO dosing ranged from 8.25% to 18.8%, whereas that of curcumin ranged from 0.152% to 1.25% under the matched vehicle conditions.

The effect of CUR-PRO on C_max_ was dependent on vehicle composition. The C_max_ of released free curcumin after CUR-PRO dosing was lower than that of curcumin in Vehicle 1 (11.6 ± 0.8 versus 40.8 ± 5.7 ng/mL), but higher in Vehicle 2 (23.3 ± 6.3 versus 4.39 ± 0.66 ng/mL) and Vehicle 3 (7.92 ± 1.12 versus 3.79 ± 0.49 ng/mL). CUR-PRO dosing also resulted in prolonged t_1/2_ and MRT_inf_ values for released curcumin compared with direct curcumin dosing. Together with the detection of intact CUR-PRO in plasma (Figure 2D; Table 2), this pattern is consistent with prolonged systemic input and gradual prodrug conversion; however, the present design does not distinguish increased absorption from reduced presystemic loss, altered clearance, or formation-limited release. Intact CUR-PRO exposure showed strong vehicle dependence, with Vehicle 2 producing the highest intact prodrug exposure (AUC_0-last_: 322 ± 154 h·ng/mL; C_max_: 260 ± 91 ng/mL). Thus, CUR-PRO increased curcumin exposure across the matched vehicle conditions tested, while both the magnitude and the kinetic basis of the enhancement remained vehicle dependent.

#### 3.3.2. Improved Exposure of Betulinic Acid Prodrug (BA-PRO)

**Figure 3:**
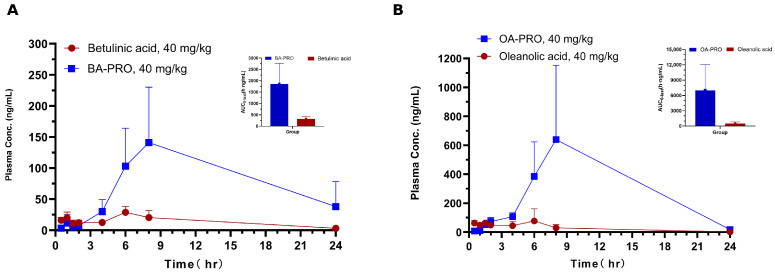
Plasma concentration–time profiles of released parent drugs after oral administration of triterpenoid prodrugs or their corresponding parent drugs. (**A**) Betulinic acid exposure after BA-PRO (40 mg/kg, equivalent to 14.2 mg/kg betulinic acid) or betulinic acid (40 mg/kg). (**B**) Oleanolic acid exposure after OA-PRO (40 mg/kg, equivalent to 14.2 mg/kg oleanolic acid) or oleanolic acid (40 mg/kg). Data are presented as mean ± SD (*n* = 3).

BA-PRO increased the oral exposure of betulinic acid after oral dosing (Figure 3A; Table 3). At a dose of 40 mg/kg (equivalent to 14.16 mg/kg betulinic acid), BA-PRO produced a mean AUC_0-last_ of 1857 ± 905 h·ng/mL and a C_max_ of 141 ± 89 ng/mL for released betulinic acid. In comparison, oral administration of free betulinic acid at 40 mg/kg yielded an AUC_0-last_ of 328 ± 100 h·ng/mL and a C_max_ of 32.1 ± 6.7 ng/mL. After dose normalization, BA-PRO increased betulinic acid exposure by 16.0-fold based on AUC_0-last_. Because intact BA-PRO was not quantified and no intravenous BA-PRO reference was available, the relative contributions of absorption, presystemic metabolism, systemic conversion, and clearance cannot be separated from these data.

#### 3.3.3. Improved Exposure of Oleanolic Acid Prodrug (OA-PRO)

OA-PRO also increased the oral exposure of oleanolic acid (Figure 3B; Table 3). At a dose of 40 mg/kg (equivalent to 14.16 mg/kg oleanolic acid), OA-PRO produced an AUC_0-last_ of 7012 ± 5030 h·ng/mL and a C_max_ of 666 ± 469 ng/mL for released oleanolic acid. In comparison, oral administration of free oleanolic acid at 40 mg/kg yielded an AUC_0-last_ of 500 ± 329 h·ng/mL and a C_max_ of 91 ± 40 ng/mL. After dose normalization, OA-PRO increased oleanolic acid exposure by 38.4-fold based on AUC_0-last_. Although inter-animal variability was evident, these results indicate increased released-parent exposure under the tested conditions. Because intact OA-PRO was not quantified and no intravenous OA-PRO reference was available, the specific kinetic determinants of the exposure increase remain unresolved.

### 3.4. Intestinal Lymphatic Transport of the Curcumin Prodrug CUR-PRO

**Figure 4:**
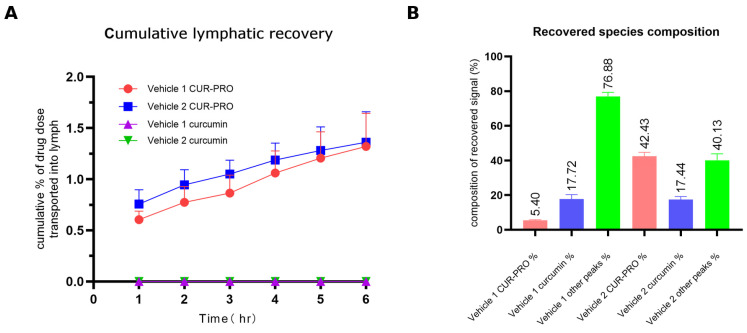
Cumulative lymphatic recovery and recovered species composition of curcumin-related 412 nm UV signals after oral administration of CUR-PRO or curcumin under matched vehicle conditions. (**A**) Cumulative lymphatic recovery over 0–6 h, expressed as a percentage of the administered curcumin-equivalent dose. (**B**) Composition of the recovered signals over 0–6 h after CUR-PRO dosing, including intact CUR-PRO, released curcumin, and unassigned peaks. Data are presented as mean ± SD (*n* = 3). Unassigned 412 nm UV peaks (other peaks) were semi-quantitatively expressed as curcumin equivalents and were not structurally confirmed.

To determine whether CUR-PRO-related material could access the intestinal lymphatic pathway, mesenteric lymph was collected after oral administration of CUR-PRO or free curcumin under two matched vehicle conditions (Table 4; Figure 4). After free curcumin dosing, no curcumin-related 412 nm UV signals were detected in lymph under the present analytical conditions. In contrast, CUR-PRO dosing generated readily detectable curcumin-related UV signals in mesenteric lymph under both vehicle conditions, demonstrating lymphatic access of CUR-PRO-related material. Representative lymph samples from CUR-PRO-dosed rats appeared yellow, whereas those from free-curcumin-dosed rats appeared clear to milky white (Supporting Information Figure S1); this visual difference was consistent with the chromatographic findings but is qualitative and should not be overinterpreted. Because total recovery was limited and several UV peaks were not structurally identified, these data do not quantify the contribution of lymphatic transport to the overall systemic exposure increase.

The overall curcumin-equivalent lymphatic recovery was comparable between Vehicle 1 and Vehicle 2 (1.32 ± 0.32% versus 1.36 ± 0.30% of the administered curcumin-equivalent dose). However, the recovered species profile differed markedly: Vehicle 2 increased the fraction of intact CUR-PRO in lymph from 5.6% to 43.1%, while the recovery of free curcumin remained low and comparable between the two vehicles (6.31 ± 1.53 and 6.63 ± 2.25 μg, respectively). These results indicate that vehicle composition influences the balance between intact prodrug and other curcumin-related UV-absorbing species recovered in lymph, without substantially altering total lymphatic recovery.

The unassigned 412 nm UV peaks were semi-quantitatively expressed as curcumin equivalents but were not structurally confirmed by MS/MS and should therefore be interpreted as UV-based signals rather than identified metabolites.

### 3.5. Stability and Conversion Behavior of CUR-PRO in Biorelevant and Biological Media

**Figure 5:**
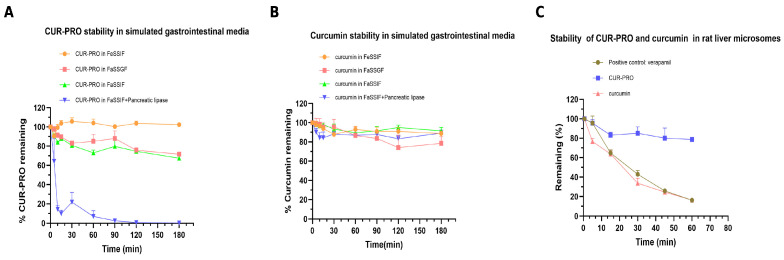
In vitro stability and enzymatic processing of CUR-PRO and curcumin. (**A**) CUR-PRO stability in simulated gastrointestinal media at 37 °C, including FaSSIF supplemented with pancreatic lipase. (**B**) Curcumin stability under the corresponding simulated gastrointestinal conditions. (**C**) Depletion profiles of CUR-PRO, curcumin, and verapamil in rat liver microsomes. Remaining percentages were calculated relative to the initial sample. Data are presented as mean ± SD where applicable; verapamil was used as a positive control to confirm metabolic activity of the microsomal incubation system.

**Figure 6:**
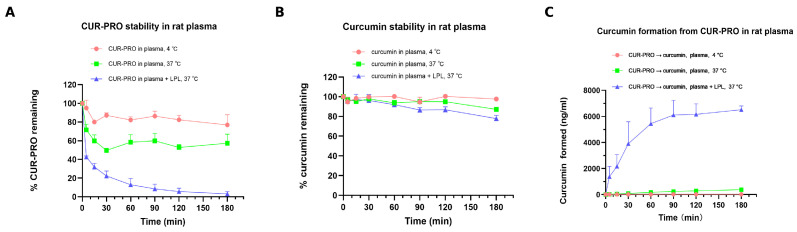
Rat plasma stability and LPL-enhanced conversion of CUR-PRO. (**A**) Stability of intact CUR-PRO in rat plasma at 4 °C, 37 °C, and 37 °C with LPL supplementation. (**B**) Stability of curcumin under the corresponding plasma conditions. (**C**) Formation of curcumin from CUR-PRO in rat plasma at 4 °C, 37 °C, and 37 °C with LPL supplementation.

To further examine the species recovered in mesenteric lymph and the subsequent conversion of CUR-PRO in biological matrices, a series of in vitro stability and conversion experiments was performed. The results are summarized in Table 5 and Figure 5 and Figure 6.

#### 3.5.1. Gastrointestinal Stability and Pancreatic Lipase-Mediated Conversion

Curcumin remained relatively stable over 180 min in FeSSIF and FaSSIF, with 88.71% and 91.59% remaining at 180 min, respectively. A greater decrease was observed in FaSSGF, where 78.54% of curcumin remained at 180 min. In FaSSIF supplemented with pancreatic lipase, curcumin showed only a modest decline over 180 min, with 89.23% remaining.

CUR-PRO displayed medium-dependent stability. In FeSSIF, no clear time-dependent depletion was observed, and the apparent remaining percentage fluctuated around the initial level. In contrast, CUR-PRO gradually decreased in FaSSGF and FaSSIF, with 71.65% and 67.50% remaining at 180 min, respectively. The most pronounced depletion occurred in FaSSIF supplemented with pancreatic lipase, where intact CUR-PRO decreased rapidly and only 0.36% remained at 180 min (Figure 5A). Free curcumin was not detected in the enzyme-free FeSSIF, FaSSIF, or FaSSGF incubations of CUR-PRO (Figure 5B). In the lipase-containing FaSSIF system, a putative curcumin monoacylglycerol-like derivative was detected, with ions at approximately *m*/*z* 585 in positive-ion mode and *m*/*z* 583 in negative-ion mode. This assignment remains tentative because the species was not confirmed using an authentic standard or definitive structural characterization. These findings suggest that CUR-PRO does not directly release free curcumin under enzyme-free simulated gastrointestinal conditions but can undergo marked depletion and conversion in pancreatic-lipase-supplemented simulated intestinal medium. This enzymatic susceptibility may help explain why vehicle composition, by altering the local digestive environment, influences the proportion of intact CUR-PRO that reaches the lymphatic compartment (Section 3.4).

#### 3.5.2. Rat Liver Microsomal Stability

In rat liver microsomes, intact CUR-PRO showed slower depletion than free curcumin. At 60 min, 78.72% of intact CUR-PRO remained, whereas only 16.42% of free curcumin remained. The estimated half-lives were 196.5 min for CUR-PRO and 23.1 min for curcumin. Verapamil, used as a positive control, was rapidly depleted with an estimated half-life of 22.3 min, confirming the metabolic activity of the incubation system (Figure 5C).

These results indicate that the intact lipid-conjugated CUR-PRO was more stable than free curcumin in the rat liver microsomal system. This observation suggests that lipid conjugation may reduce the immediate microsomal depletion of the curcumin moiety before release of the parent drug. Together with the in vivo pharmacokinetic results, these findings support the potential contribution of lipid conjugation to improved curcumin exposure after oral administration of CUR-PRO.

#### 3.5.3. Rat Plasma Stability and LPL-Enhanced Conversion

In rat plasma, curcumin remained relatively stable at 4 °C, with 97.66% remaining at 180 min. At 37 °C, curcumin showed a greater time-dependent decrease, with 87.12% remaining at 180 min. LPL supplementation did not further reduce curcumin stability, which remained comparable to that observed in plasma at 37 °C without LPL.

CUR-PRO showed more pronounced temperature- and enzyme-dependent conversion in rat plasma. At 4 °C, 76.96% of CUR-PRO remained at 180 min, whereas 57.40% remained at 37 °C. When LPL was added to rat plasma at 37 °C, intact CUR-PRO decreased rapidly, with only 3.20% remaining at 180 min (Figure 6A,B). Consistent with this depletion, released curcumin was detected in CUR-PRO incubation samples. Curcumin formation was limited at 4 °C (24.3 ng/mL at 180 min), increased at 37 °C (378.7 ng/mL at 180 min), and was markedly enhanced in the presence of LPL (6513 ng/mL at 180 min; Figure 6C).

These results indicate that CUR-PRO is relatively protected against immediate depletion under low-temperature plasma handling conditions but can undergo conversion in rat plasma at physiological temperature. Exogenous LPL supplementation markedly accelerated CUR-PRO depletion and curcumin formation under the tested in vitro conditions. This finding supports the ability of lipolytic enzymes to promote CUR-PRO conversion, but it does not establish endogenous circulating LPL as the dominant conversion enzyme in vivo or demonstrate that systemic conversion occurs exclusively after lymphatic transport.

Overall, the in vitro data are consistent with a sequential processing pathway in which CUR-PRO is susceptible to pancreatic lipase-mediated digestion, displays greater microsomal stability than free curcumin, and undergoes plasma conversion that is accelerated by LPL. These findings provide qualitative mechanistic context for the in vivo observations, but they do not establish a quantitative in vitro–in vivo correlation or identify the rate-limiting step governing systemic exposure.

### 3.6. Discussion

This study provides proof-of-concept evidence that 1,3-diolein-based lipidation can improve the oral exposure of selected poorly water-soluble natural products under the experimental conditions tested. The current evidence, however, does not establish that lymphatic transport is the sole or dominant cause of the exposure increase. Matched-vehicle comparisons reduce the likelihood that the findings are attributable to one specific formulation, but they cannot quantitatively separate contributions from dissolution or dispersion, intestinal permeability, presystemic metabolism, systemic disposition, prodrug conversion, and lymphatic transport. The lymph study demonstrates that CUR-PRO-related material reached mesenteric lymph, whereas free curcumin did not generate detectable lymphatic UV signals. Nevertheless, the approximately 1.3% total curcumin-equivalent recovery, the presence of unassigned UV peaks, and the qualitative nature of the sample-appearance observation mean that these findings should be interpreted as evidence of lymphatic access rather than quantitative proof that lymphatic targeting accounts for the systemic exposure enhancement.

The pharmacokinetic changes appear to reflect a composite process rather than a single mechanism. Detection of intact CUR-PRO in plasma and mesenteric lymph indicates that at least a fraction of the conjugate can be absorbed without complete release of curcumin in the intestinal lumen, which is consistent with uptake through lipid-processing pathways rather than only passive diffusion of the parent drug. CUR-PRO increased dose-normalized AUC across three vehicle conditions, whereas the direction and magnitude of the C_max_ change were vehicle dependent. The prolonged MRT_inf_ and t_1/2_ of released curcumin after CUR-PRO dosing are consistent with sustained systemic input and may be compatible with partial avoidance of hepatic first-pass metabolism and/or formation-limited kinetics. Improved microsomal stability of intact CUR-PRO suggests reduced immediate hepatic depletion of the conjugate, whereas plasma conversion that was accelerated by exogenous LPL in vitro, together with the delayed T_max_, supports gradual systemic release of curcumin. These observations remain preliminary and are not diagnostic of any single mechanism. Because intravenous CUR-PRO data, portal-vein exposure, complete mass-balance measurements, and separate clearance estimates for prodrug-derived curcumin were not available, the present study cannot determine whether the increased AUC resulted primarily from enhanced absorption, reduced presystemic loss, altered clearance, or conversion-limited release.

The in vitro experiments provide a qualitative sequence that can guide future mechanistic analysis: luminal lipase-mediated digestion, uptake of intact and/or processed prodrug species, access to mesenteric lymph, subsequent systemic conversion of the conjugate, and relative protection of the conjugated curcumin moiety from microsomal depletion. Exogenous LPL markedly accelerated conversion in rat plasma in vitro, but the present study does not establish that circulating LPL is the dominant enzyme responsible for prodrug conversion in vivo. These components have not been integrated into a quantitative in vitro-in vivo correlation or mechanistic pharmacokinetic model. Establishing such a model would require studies across multiple dose levels, enzyme kinetic parameters, compartmental modeling and sensitivity analysis, and time-resolved measurements of intact prodrug, intermediate species, and released parent drug in the intestinal lumen, enterocytes, portal blood, lymph, and systemic plasma, together with mass-balance information. The proposed pathway should therefore be regarded as a hypothesis-generating framework rather than a validated quantitative model.

Beyond pharmacokinetic performance, the practical utility of triglyceride-mimetic prodrugs will depend on safety and developability. A relevant translational precedent is the recent report on the oral allopregnanolone triglyceride-mimetic prodrug GlyphAllo (SPT-300), which progressed from preclinical lymphatic-transport and tissue-distribution studies to early clinical evaluation and was reported to be generally well tolerated [32]. This experience supports the translational feasibility of the general strategy but cannot be extrapolated directly to CUR-PRO, BA-PRO, or OA-PRO because the parent drugs, linker structures, metabolic products, doses, and intended treatment settings differ. Accordingly, repeated-dose studies will be required to assess chronic tolerability, tissue distribution, potential accumulation of lipid-conjugated species, and the safety of promoiety- and linker-derived metabolites. Developability assessment should also address scalable synthesis of IN-4 and the final prodrugs, control of regio- and stereochemical composition, residual reagents and catalysts, batch-to-batch reproducibility, chemical and formulation stability, drug loading, and oral dosage-form manufacturability. Because these studies were not performed in the present proof-of-concept work, these considerations are presented as priorities for future investigation rather than established attributes of the current prodrug series.

Compared with previously reported triglyceride-mimetic prodrug systems [28,29], the present work provides a complementary evaluation in selected natural products by combining cross-compound exposure assessment, matched-vehicle pharmacokinetics, lymphatic sampling, and enzymatic profiling; it does not establish superiority over existing systems. Other limitations include the small sample size, the inability to quantify intact BA-PRO and OA-PRO, the qualitative or semi-quantitative nature of the lymph analysis, and the absence of physicochemical thresholds or structure-platform relationships. Overall, CUR-PRO serves as a mechanistic model showing that lymphatic access and sequential enzymatic processing are plausible contributors to enhanced exposure, while their quantitative contribution relative to other absorption and disposition mechanisms remains unresolved. Dedicated intervention studies that selectively suppress chylomicron-mediated lymphatic transport, together with portal-vein sampling and compartmental pharmacokinetic modeling, will be required to separate and quantify these pathway contributions.

## 4. Conclusions

In this study, a 1,3-diolein-based lipid–drug conjugate strategy was developed and applied to three selected natural products spanning a polyphenol (curcumin) and two pentacyclic triterpenoids (betulinic acid and oleanolic acid). The common triglyceride-mimetic promoiety enabled the synthesis of three representative prodrugs, CUR-PRO, BA-PRO, and OA-PRO, through modular convergent routes.

In exploratory rat pharmacokinetic studies, all three prodrugs increased systemic exposure to the corresponding released parent-drug analytes under the tested conditions. For CUR-PRO, exposure increased across three vehicle systems, while intact CUR-PRO was detected in plasma with vehicle-dependent exposure. Mesenteric lymph sampling showed detectable CUR-PRO-related 412 nm UV signals, including intact CUR-PRO, whereas free curcumin did not generate detectable curcumin-related lymphatic signals under the tested conditions. These findings support lymphatic access of CUR-PRO-related material but do not quantify the fraction of systemic exposure attributable to that pathway.

The vehicle-dependent recovery of intact CUR-PRO in lymph indicates that vehicle composition may affect the species profile of the prodrug in the lymphatic compartment. Although the small sample size limits definitive mechanistic conclusions, the in vitro observations of pancreatic lipase-mediated digestion and LPL-enhanced conversion raise the hypothesis that vehicle composition may modulate local enzymatic processing of the prodrug before or during lymphatic uptake, thereby influencing the fraction of intact conjugate that reaches the lymph.

For BA-PRO and OA-PRO, the observed increases in released parent-drug exposure (16.0- and 38.4-fold, respectively) provide preliminary support for the potential applicability of this lipidation strategy to selected poorly water-soluble natural products, although intact prodrug quantification and detailed conversion pathways require further investigation.

Collectively, these findings provide a proof-of-concept foundation for further evaluation of triglyceride-mimetic lipid prodrugs for selected poorly water-soluble natural products. The lymphatic transport data provide qualitative evidence supporting lymphatic access of CUR-PRO, although the quantitative contribution of this pathway to the overall exposure increase remains to be established. Further studies using a broader and more diverse compound set will be needed to establish structure-platform relationships and broader generalizability.

## Figures and Tables

**Table 1:** Pharmacokinetic parameters of curcumin exposure after oral administration of CUR-PRO or curcumin under matched vehicle conditions in male Sprague–Dawley rats.

Test	Vehicle/ Administration	Dose (mg/kg)	C_max_ (ng/mL)	AUC_0-last_/AUC_0-∞_ (h·ng/mL)	t_1/2_/MRT_inf_ (h)	F (%)	AUC_0-last_ Fold
CUR-PRO	Vehicle 1/po	40 (13.24 equiv.)	11.6 ± 0.8	105 ± 7.0/128 ± 14	10.7 ± 2.9/ 13.1 ± 3.4	18.8	15.0
Curcumin	Vehicle 1/po	40	40.8 ± 5.7	20.9 ± 1.3/22.8 ± 1.1	0.406 ± 0.071/ 0.616 ± 0.022	1.25	-
CUR-PRO	Vehicle 2/po	40 (13.24 equiv.)	23.3 ± 6.3	90.5 ± 9.5/109 ± 7.8	10.1 ± 0.7/ 12.1 ± 1.9	16.3	70.9
Curcumin	Vehicle 2/po	40	4.39 ± 0.66	3.86 ± 0.24/5.13 ± 0.42	0.71 ± 0.096/1.13 ± 0.12	0.230	-
CUR-PRO	Vehicle 3/po	40 (13.24 equiv.)	7.92 ± 1.12	45.9 ± 1.8/48.2 ± 1.8	5.66 ± 0.60/ 6.76 ± 0.78	8.25	54.3
Curcumin	Vehicle 3/po	40	3.79 ± 0.49	2.56 ± 0.17/2.89 ± 0.29	0.46 ± 0.07/0.76 ± 0.11	0.152	-
Curcumin	Vehicle 4/iv	4	1299 ± 476 (C_0_)	168 ± 48/172 ± 46	1.38 ± 0.81/0.60 ± 0.39	100	-

Data are presented as mean ± SD (*n* = 3). For the CUR-PRO groups, C_max_ and AUC values refer to the exposure of the released parent drug, free curcumin. Vehicle 1: PEG 400/Transcutol HP/Solutol HS 15/0.9% saline (30:10:7:53, *v*/*v*/*v*/*v*); Vehicle 2: oleic acid/Tween 80/0.9% saline (2:1.25:96.75, *v*/*v*/*v*); Vehicle 3: 0.5% CMC-Na; Vehicle 4: DMSO/ethanol/propylene glycol/PEG 400/0.9% saline (0.5:20:10:40:30, *v*/*v*/*v*/*v*/*v*).

**Table 2:** Pharmacokinetic parameters of intact CUR-PRO after oral administration of CUR-PRO under matched vehicle conditions in male Sprague–Dawley rats.

Test	Vehicle/ Administration	Dose (mg/kg)	C_max_ (ng/mL)	AUC_0-last_/AUC_0-∞_ (h·ng/mL)	t_1/2_/MRT_inf_ (h)
CUR-PRO	Vehicle 1/po	40 (13.24 equiv.)	5.82 ± 1.76	48.9 ± 21.3/50.4 ± 21.6	4.25 ± 0.41/ 7.62 ± 0.61
CUR-PRO	Vehicle 2/po	40 (13.24 equiv.)	260 ± 91	322 ± 154/327 ± 155	2.06 ± 0.67/ 1.27 ± 0.03
CUR-PRO	Vehicle 3/po	40 (13.24 equiv.)	12.0 ± 1.12	23.5 ± 2.0/26.9 ± 2.8	8.93 ± 1.10/ 9.61 ± 1.61

Data are presented as mean ± SD (*n* = 3). Cmax and AUC values refer to the exposure of intact CUR-PRO. Vehicle compositions were the same as those described in Table 1.

**Table 3:** Pharmacokinetic parameters of betulinic acid and oleanolic acid exposure after oral administration of BA-PRO, OA-PRO, or their corresponding parent drugs in male Sprague–Dawley rats. For prodrug-dosed groups, the reported values refer to exposure of the released parent drug.

Compound	Dose (mg/kg)	C_max_ (ng/mL)	AUC_0-last_ (h·ng/mL)	AUC_0-∞_ (h·ng/mL)	t_1/2_ (h)	MRT_inf_ (h)	AUC_0-Last_ Fold
BA-PRO	40 (14.16 equiv.)	141 ± 89	1857 ± 905	2952 ± 1797	13.7 ± 9.9	23.5 ± 14.1	16.0
Betulinic Acid PO	40	32.1 ± 6.7	328 ± 100	351 ± 123	7.20 ± 2.64	11.2 ± 2.5	-
OA-PRO	40 (14.16 equiv.)	666 ± 469	7012 ± 5030	7183 ± 4850	4.82 ± 4.03	10.6 ± 4.7	38.4
Oleanolic Acid PO	40	91 ± 40	500 ± 329	794 ± 50	6.58 ± 2.91	10.8 ± 6.0	-

**Table 4:** Summary of 0–6 h mesenteric lymphatic recovery after oral administration of CUR-PRO or curcumin under matched vehicle conditions. Data are presented as mean ± SD (*n* = 3).

Vehicle	Treatment	Total Lymph Volume (mL)	Intact CUR-PRO (μg Equation)	Free Curcumin (μg)	Unassigned 412 nm Peaks (μg Equation)	Total Recovery (μg Equation)	% Dose Recovered in Lymph
Vehicle 1	CUR-PRO	1.84 ± 0.62	2.05 ± 1.00	6.31 ± 1.53	28.53 ± 10.44	36.88 ± 12.59	1.32 ± 0.32
Vehicle 1	Curcumin	1.61 ± 0.34	ND	ND	ND	ND	ND
Vehicle 2	CUR-PRO	2.05 ± 0.78	16.17 ± 5.42	6.63 ± 2.25	14.74 ± 2.58	37.54 ± 9.73	1.36 ± 0.30
Vehicle 2	Curcumin	2.18 ± 0.55	ND	ND	ND	ND	ND

Note: CUR-PRO amounts were converted to curcumin equivalents using the molecular-weight ratio. Unassigned 412 nm peaks represent the sum of additional UV-absorbing peaks between approximately 3.0 and 30.0 min after excluding the verified curcumin and CUR-PRO peaks; these signals were semi-quantitatively expressed as curcumin equivalents and were not structurally confirmed by MS/MS. ND, not detected under the present analytical conditions.

**Table 5:** Summary of CUR-PRO and curcumin stability/conversion in simulated gastrointestinal and biological media.

System	Condition	Curcumin Remaining	CUR-PRO Remaining	Main Observation
Simulated GI media	FeSSIF, 37 °C, 180 min	88.71%	102.34%	CUR-PRO showed no apparent depletion in FeSSIF.
Simulated GI media	FaSSIF, 37 °C, 180 min	91.59%	67.50%	CUR-PRO gradually decreased in FaSSIF, with no detectable free curcumin.
Simulated GI media	FaSSGF, 37 °C, 180 min	78.54%	71.65%	Both compounds showed partial loss under gastric simulated conditions.
Lipase-containing medium	FaSSIF + pancreatic lipase, 37 °C, 180 min	89.23%	0.36%	CUR-PRO was rapidly depleted; a curcumin monoglyceride-like derivative was observed.
Rat liver microsomes	NADPH, 37 °C, 60 min	16.42%	78.72%	CUR-PRO was more stable than free curcumin in microsomes.
Rat plasma	4 °C, 180 min	97.66%	76.96%	Low temperature reduced conversion/depletion.
Rat plasma	37 °C, 180 min	87.12%	57.40%	CUR-PRO showed time-dependent depletion and curcumin formation.
Rat plasma + LPL	37 °C, 180 min	77.81%	3.20%	LPL markedly accelerated CUR-PRO depletion and curcumin formation.

## Data Availability

All data supporting the findings of this study are contained within the article and its Supporting Information.

## Supplementary Materials

The following supporting information can be downloaded at https://www.mdpi.com/article/10.3390/pharmaceutics18070899/s1. Supporting Information S1: LC–MS/MS method validation for curcumin in rat plasma; Table S1: Summary of validation results for the LC-MS/MS method for curcumin in rat plasma; Figure S1: Representative photographs of mesenteric lymph samples collected after oral administration of CUR-PRO or curcumin under matched vehicle conditions; Figure S2: Representative HPLC-UV chromatograms of mesenteric lymph samples at 412 nm after oral administration of CUR-PRO or curcumin under matched vehicle conditions; Figure S3: Chemical characterization data for IN-4; Figure S4: Chemical characterization data for CUR-PRO; Figure S5: Chemical characterization data for BA-PRO; Figure S6: Chemical characterization data for OA-PRO.
