# Supplementary material for: Enhancing Oral Bioavailability of Poorly Water-Soluble Natural Products via Lipid–Drug Conjugates
Source: Pharmaceutics. 2026 Jul 22;18(7):899. doi: 10.3390/pharmaceutics18070899 (PMC13416388; doi:10.3390/pharmaceutics18070899)

Supporting Information

Enhancing Oral Bioavailability of Poorly Water-Soluble Natural Products via Lipid-Drug Conjugates

S1. Analytical Method Validation for Curcumin in Rat Plasma

The LC-MS/MS method used for the quantification of curcumin in rat plasma was validated in accordance with commonly accepted bioanalytical method validation principles, including assessments of selectivity, calibration curve, lower limit of quantification (LLOQ), accuracy, precision, recovery, and matrix effect. The validation results are summarized in Table S1.

Selectivity.

Selectivity was evaluated by analyzing blank plasma samples obtained from at least six individual rats, blank plasma samples spiked with curcumin at the LLOQ level, and post-dose plasma samples. No significant endogenous interference was observed at the retention times of curcumin or the internal standard.

Calibration curve and linearity.

Calibration standards were prepared by spiking blank rat plasma with curcumin to yield final concentrations ranging from 1 to 5000 ng/mL. Calibration curves were constructed by plotting the peak-area ratio of curcumin to the internal standard against the nominal concentration using weighted ($1/x^2$) linear regression. The method showed good linearity over the tested concentration range, with correlation coefficients (R^2) ranging from 0.9918 to 0.9999.

Lower limit of quantification (LLOQ).

The LLOQ of curcumin in rat plasma was established at 1 ng/mL, at which the signal-to-noise ratio exceeded 10. At the LLOQ, the accuracy bias was 19.3% and the precision was 10.5%, meeting the commonly accepted criteria for quantitative bioanalysis.

Accuracy and precision.

Intra-day and inter-day accuracy and precision were assessed using QC samples at three concentration levels (low, medium, and high QC levels at 100, 500, and 2000 ng/mL, respectively), while the LLOQ was evaluated separately. Intra-day accuracy ranged from 2.9% to 7.4%, whereas inter-day accuracy ranged from 4.8% to 12.8%. Intra-day precision, expressed as relative standard deviation (RSD), ranged from 0.86% to 10.5%, and inter-day precision

ranged from 1.78% to 8.4%. These values were all within the acceptable limits for bioanalytical method validation.

Recovery.

Extraction recovery of curcumin was evaluated by comparing the peak areas obtained from plasma samples spiked before extraction with those from blank plasma extracts spiked after extraction at the corresponding concentrations (low, medium, and high QC levels). The apparent recovery of curcumin was 90.7%, 88.8%, and 97.6% at the low, medium, and high QC levels, respectively. The precision of the recovery assessment ranged from 2.4% to 11.2%, indicating acceptable reproducibility.

Matrix effect.

The matrix effect was evaluated using plasma obtained from multiple individual rats at the low, medium, and high QC levels. The mean accuracy bias (%Bias) ranged from -6.0% to 9.3%, and the coefficient of variation (%CV) ranged from 1.02% to 9.31% across the tested QC levels. These results met the predefined acceptance criteria (%Bias within ±15.0% and %CV ≤ 15.0%), indicating that matrix effects were negligible under the validated conditions.

Table S1. Summary of validation results for the LC-MS/MS method for curcumin in rat plasma.

Validation item	Condition / Level	Result	Acceptance criteria
Selectivity	Blank plasma from 6 individual rats; blank plasma spiked at the LLOQ; post-dose plasma samples	No significant endogenous interference at the retention times of curcumin or the internal standard	No significant interference
Calibration curve and linearity	1-5000 ng/mL; weighted ($1/x^2$) linear regression	R^2 = 0.9918-0.9999	R^2 > 0.99
LLOQ	1 ng/mL	S/N > 10; accuracy bias = 19.3%; precision = 10.5%	S/N > 10; accuracy within ±20% and precision ≤ 20%
Accuracy and precision	Low QC / Medium QC / High QC	Intra-day accuracy: 2.9-7.4%; Inter-day accuracy: 4.8-12.8%; Intra-day precision: 0.86-10.5%; Inter-day precision: 1.78-8.4%	Accuracy within ±15% and precision ≤ 15%
Recovery	Low QC / Medium QC / High QC	Apparent recovery of curcumin: 90.7%, 88.8%, and 97.6%; precision: 2.4-11.2%	Consistent and reproducible; precision ≤ 15%
Matrix effect	Low QC / Medium QC / High QC	%Bias: -6.0-9.3%; %CV: 1.02-9.31%	%Bias within ±15.0%; %CV ≤ 15.0%

Abbreviations: LLOQ, lower limit of quantification; QC, quality control; S/N, signal-to-noise ratio; R^2, correlation coefficient; %Bias, mean accuracy bias; %CV, coefficient of variation.

S2. Representative Lymph Sample Appearance and HPLC-UV Chromatograms

S2.1. Representative lymph sample appearance

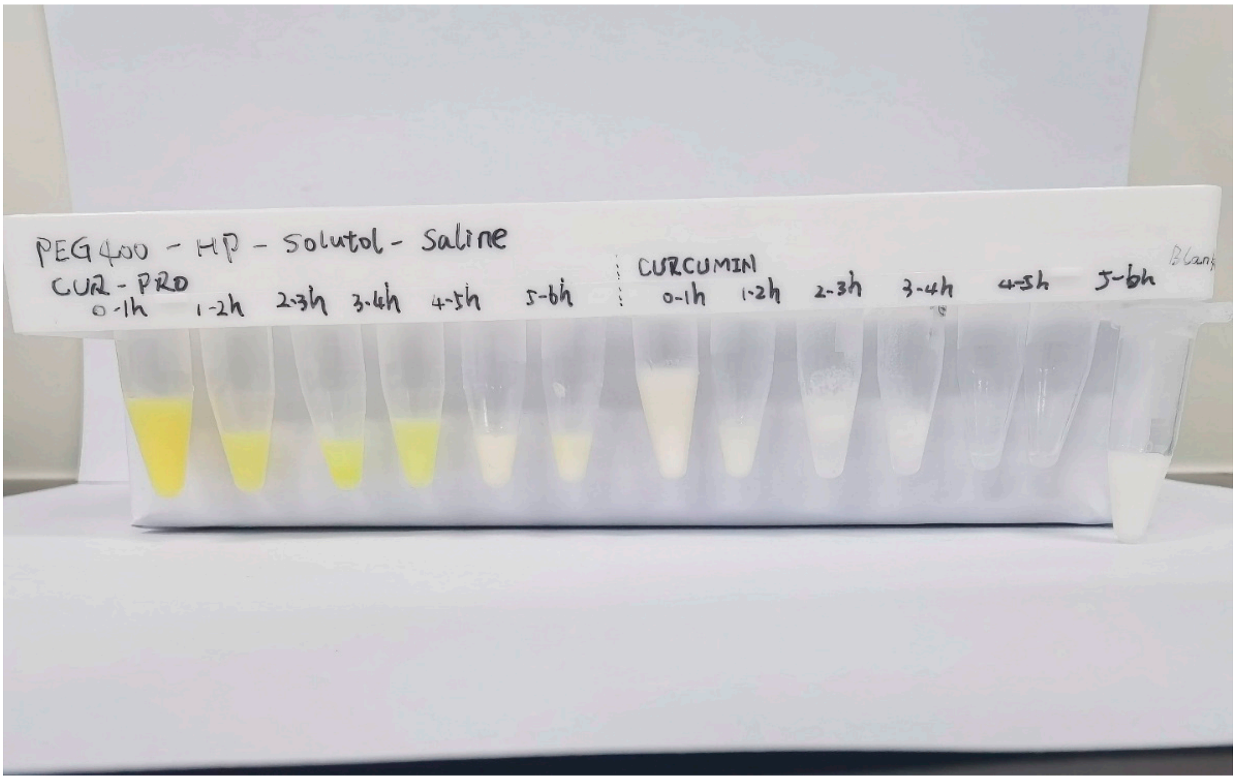

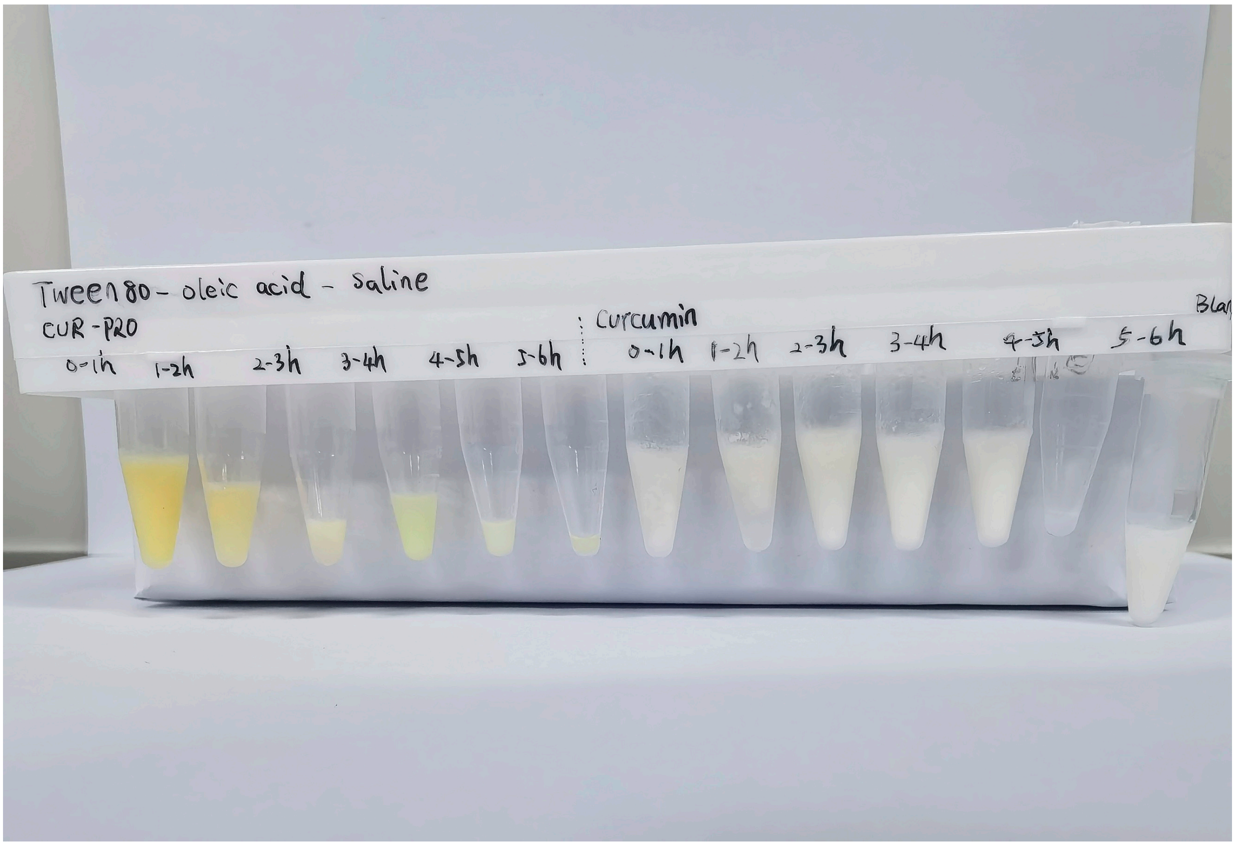

Figure S1. Representative lymph sample appearance after oral administration of CUR-PRO or curcumin under matched vehicle conditions.

Upper panel: Representative appearance of lymph samples collected at hourly intervals from 0 to 6 h after dosing under Vehicle 1. The six tubes on the left show lymph samples collected from the CUR-PRO-dosed group, which displayed a distinct yellow color, suggesting the presence of curcumin-related components. The six tubes on the right show lymph samples collected from the curcumin-dosed group over the same time intervals, which appeared colorless to milky white and were similar in appearance to the blank lymph sample shown on the far right.

Lower panel: Representative appearance of lymph samples collected at hourly intervals from 0 to 6 h after dosing under Vehicle 2. The six tubes on the left show lymph samples collected from the CUR-PRO-dosed group, which also displayed a curcumin-related yellow color. The six tubes on the right show lymph samples collected from the curcumin-dosed group over the same time intervals, which appeared colorless to milky white and were similar in appearance to the blank lymph sample shown on the far right, consistent with their HPLC-UV chromatographic profiles.

S2.2. Representative HPLC-UV chromatograms

Representative HPLC-UV chromatograms at 412 nm are shown below for blank lymph, reference standards, and representative lymph samples collected after oral administration of curcumin or CUR-PRO under matched vehicle conditions.

(A) Blank lymph

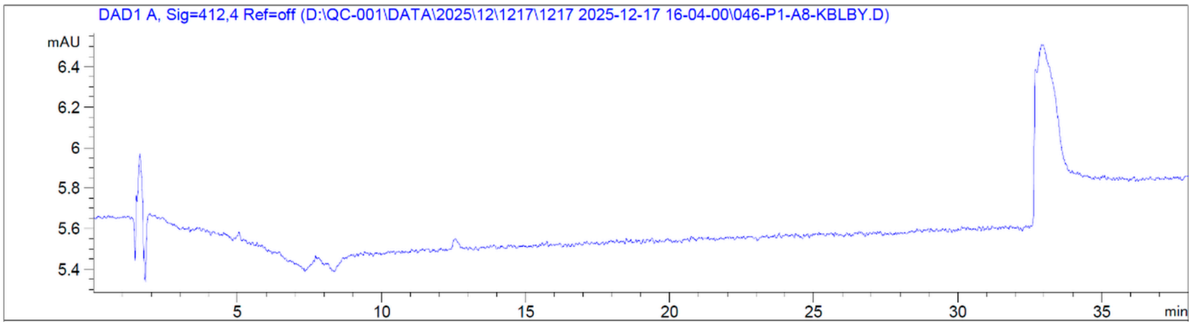

(B) Curcumin standard (RT 5.059 min)

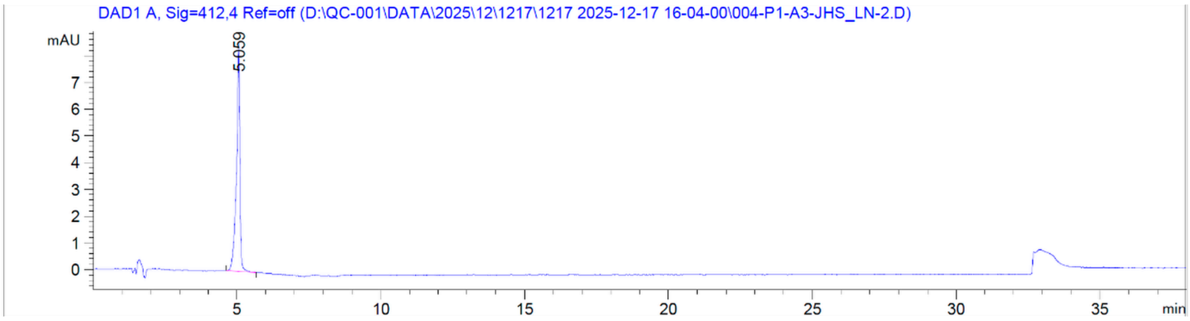

(C) CUR-PRO standard (N102C-028; RT 24.544 min)

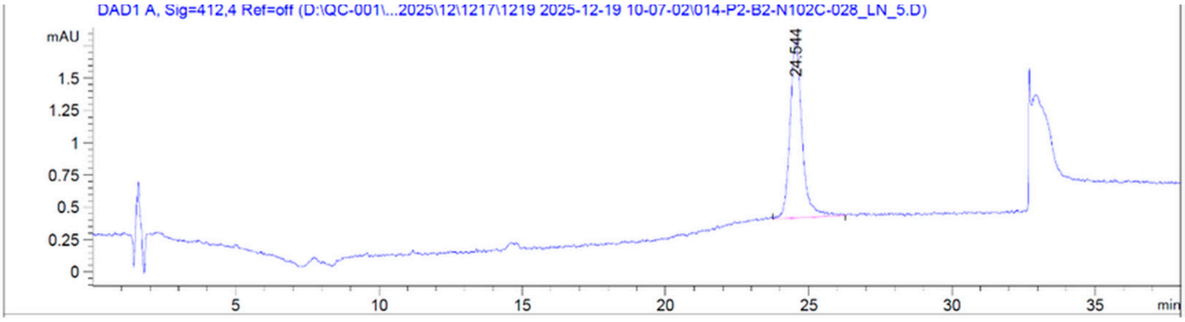

(D) Representative lymph sample chromatogram after oral administration of curcumin in Vehicle 1

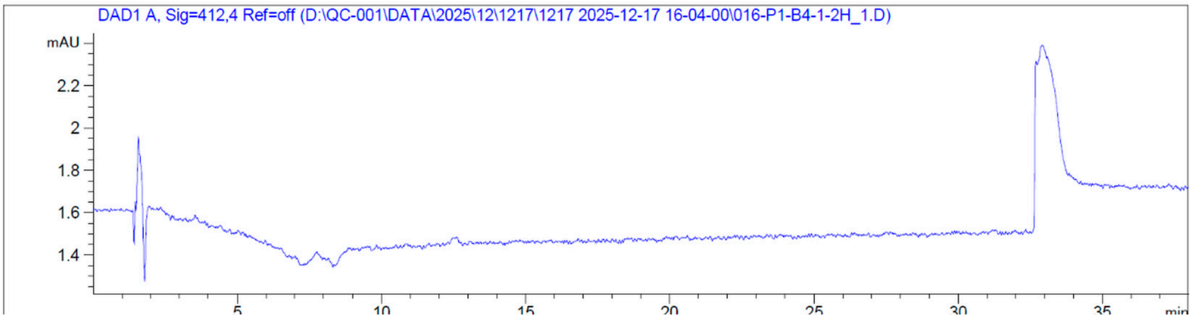

(E) Representative lymph sample chromatogram after oral administration of curcumin in Vehicle 2

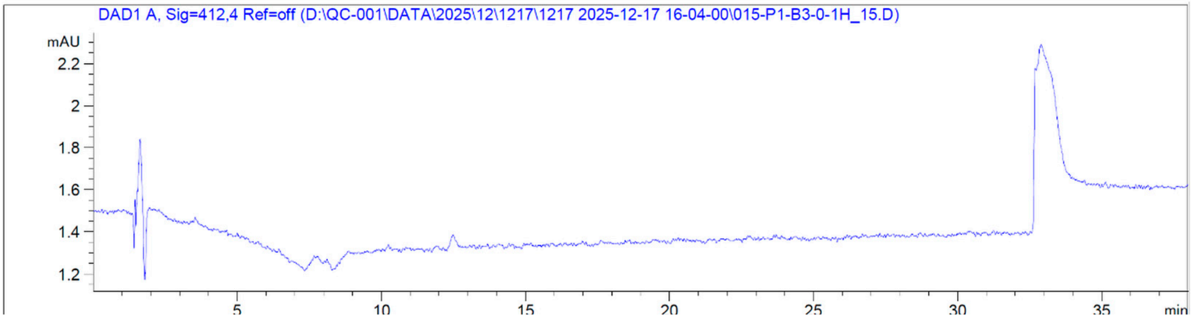

(F) Representative lymph sample chromatogram after oral administration of CUR-PRO in Vehicle 1

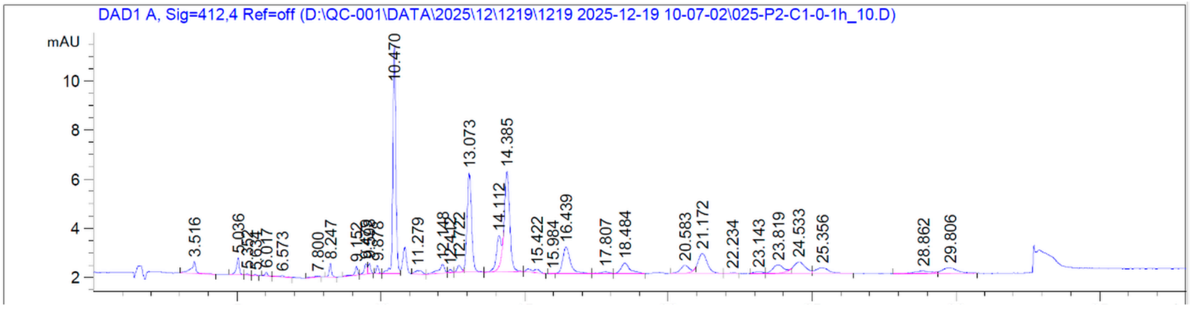

(G) Representative lymph sample chromatogram after oral administration of CUR-PRO in Vehicle 2

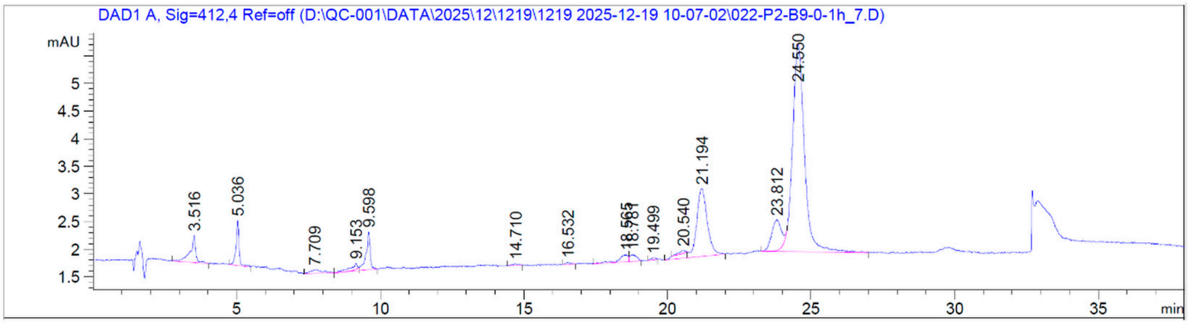

Figure S2. Representative HPLC-UV chromatograms of lymph samples and reference standards at 412 nm. (A) Blank lymph; (B) curcumin standard; (C) CUR-PRO standard (N102C-028); (D,E) representative lymph sample chromatograms after oral administration of curcumin in Vehicle 1 and Vehicle 2, respectively; and (F,G) representative lymph sample chromatograms after oral administration of CUR-PRO in Vehicle 1 and Vehicle 2, respectively.

S3. Chemical Characterization Data for Lipid Promoiety and Final Prodrugs

Figure S3. Characterization data for IN-4.

^{1}H NMR (400 MHz, $CDCl_3$)

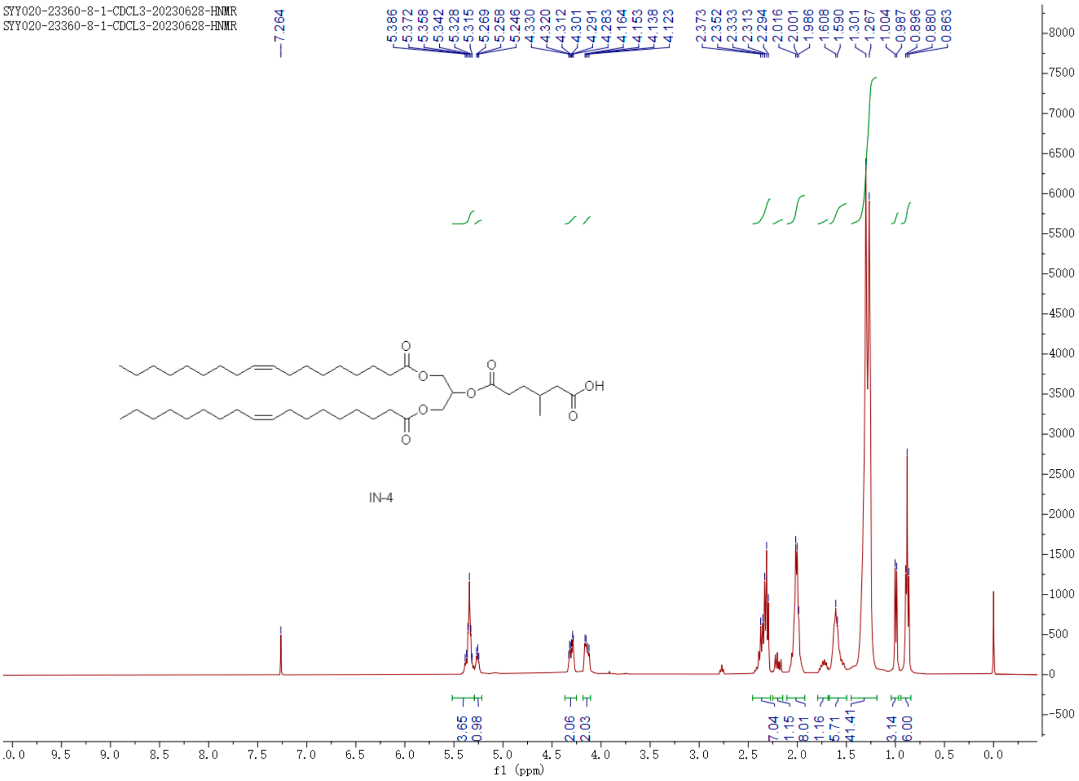

Figure S4. Characterization data for CUR-PRO.

^{1}H NMR (400 MHz, $CDCl_3$)

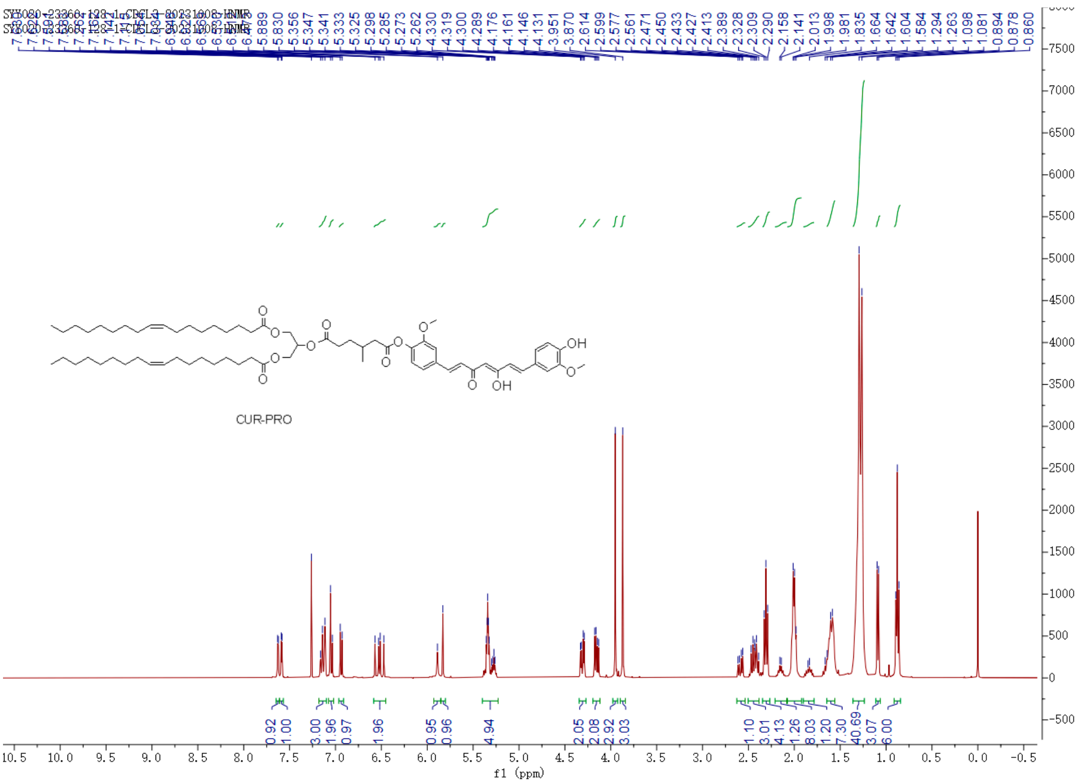

^{13}C NMR (100 MHz, $CDCl_3$)

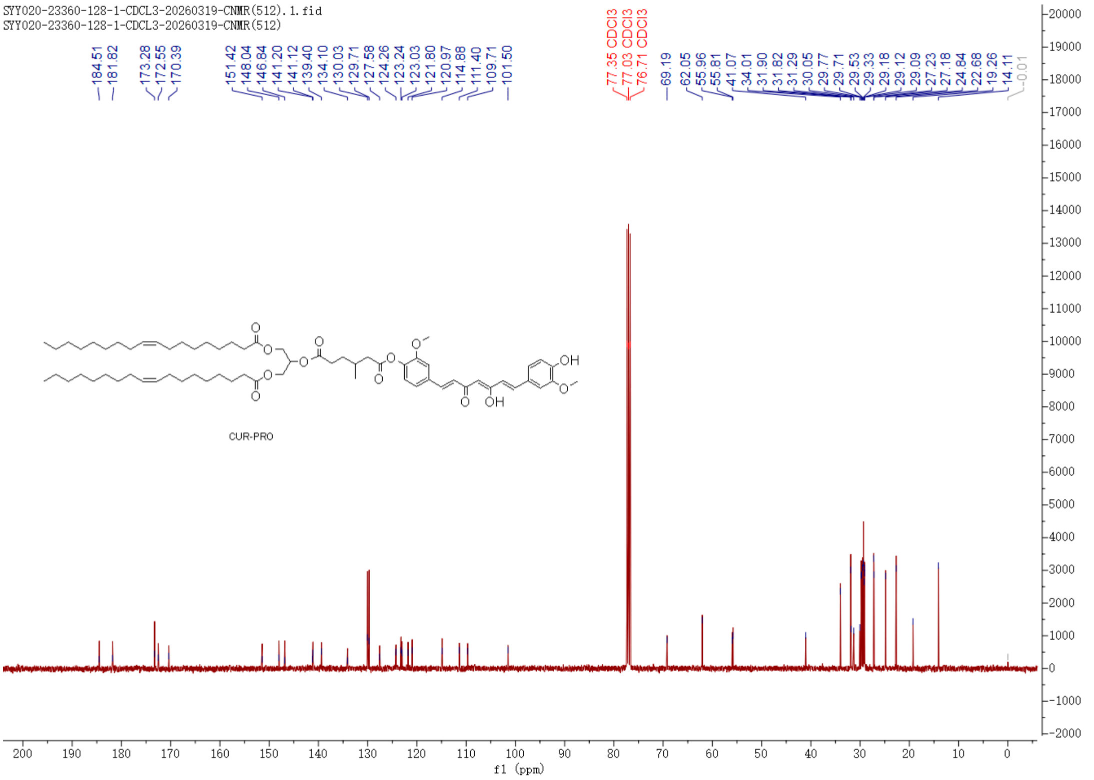

CUR-PRO TOF-MS (ESI−) *m/z* 1111.7510 [M − H]−

Sample Chromatograms

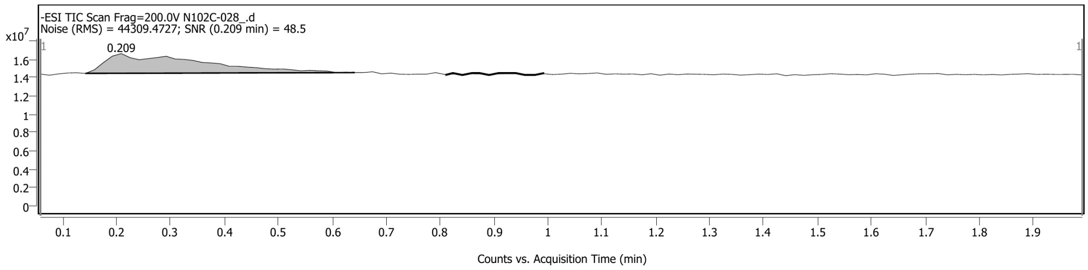

Sample Spectra

- Scan (rt: 0.159-0.558 min) Sub Peak 1 from - TIC Scan

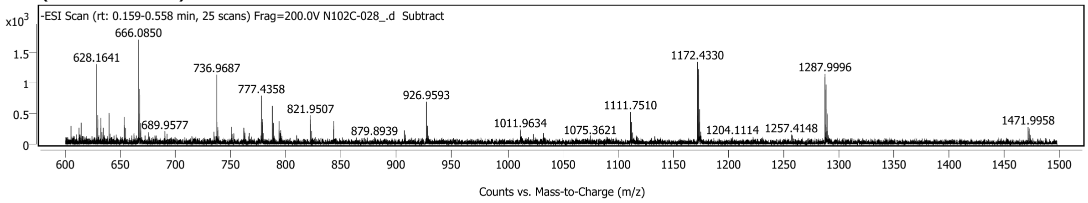

Figure S5. Characterization data for BA-PRO.

^{1}H NMR (400 MHz, $CDCl_3$)

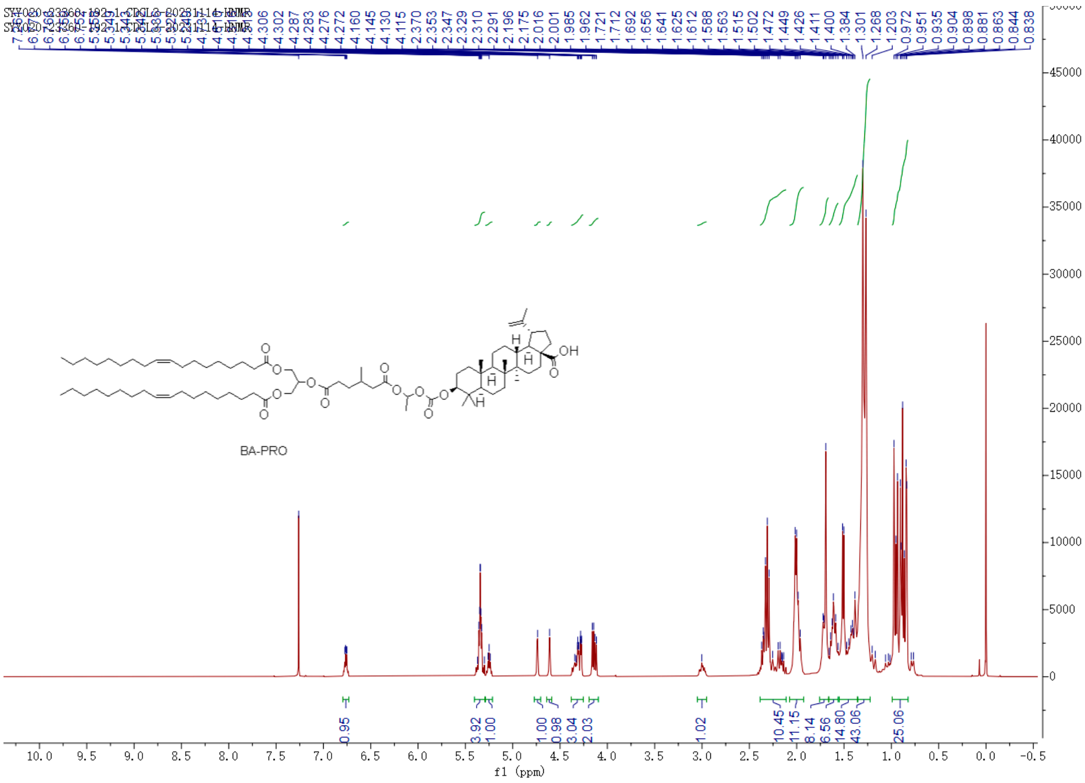

^{13}C NMR (100 MHz, $CDCl_3$)

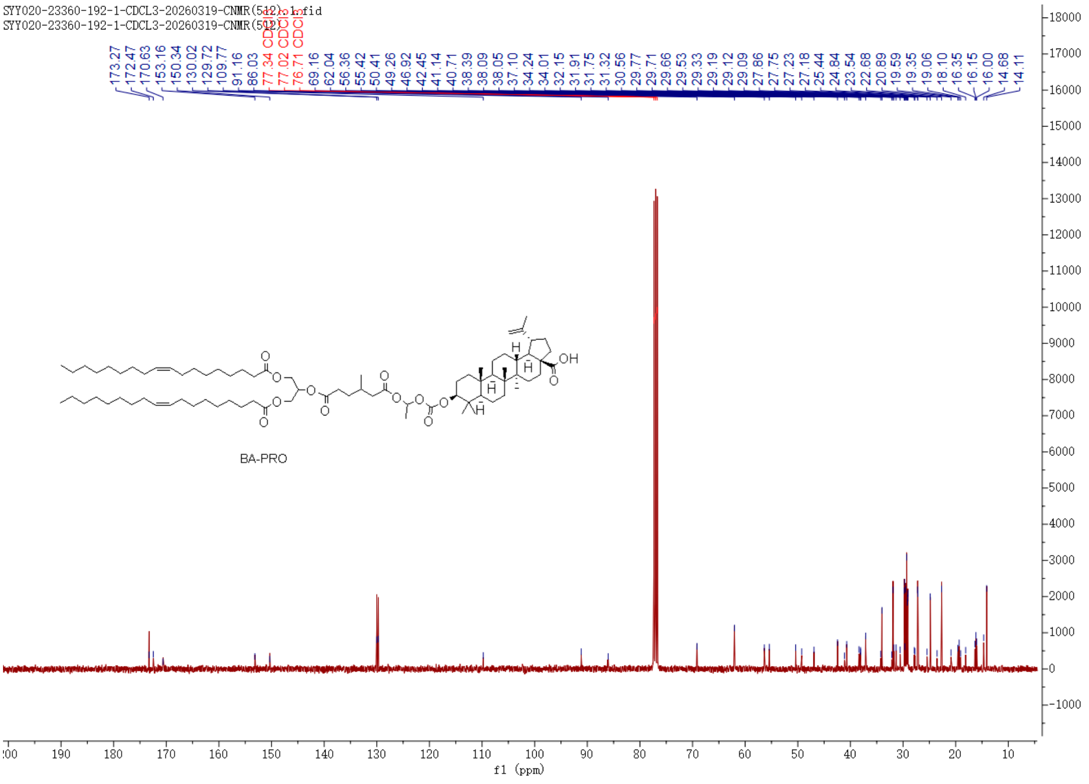

BA-PRO TOF-MS (ESI−): *m/z* 1287.9970 [M − H]−

Sample Chromatograms

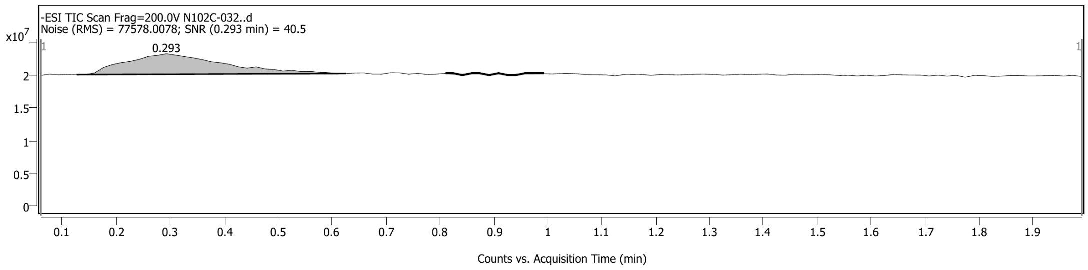

Sample Spectra

- Scan (rt: 0.176-0.559 min) Sub   Peak 1 from - TIC Scan

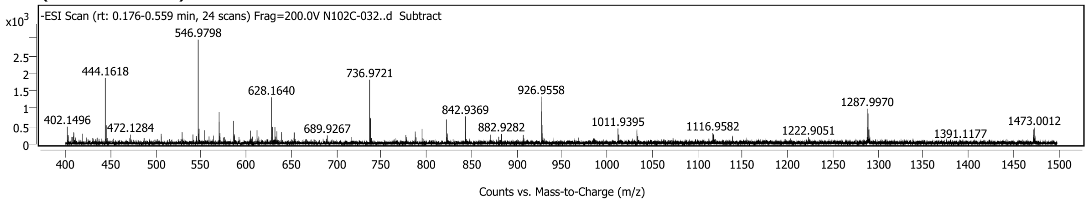

Figure S6. Characterization data for OA-PRO.

^{1}H NMR (400 MHz, $CDCl_3$)

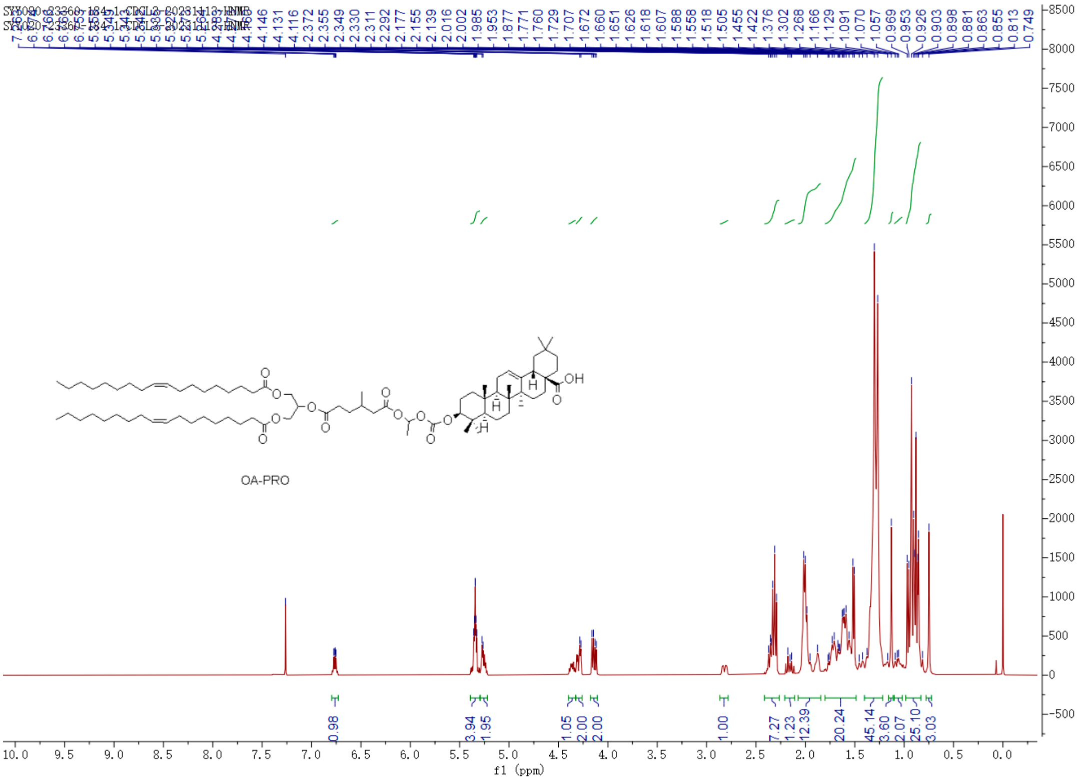

^{13}C NMR (100 MHz, $CDCl_3$)

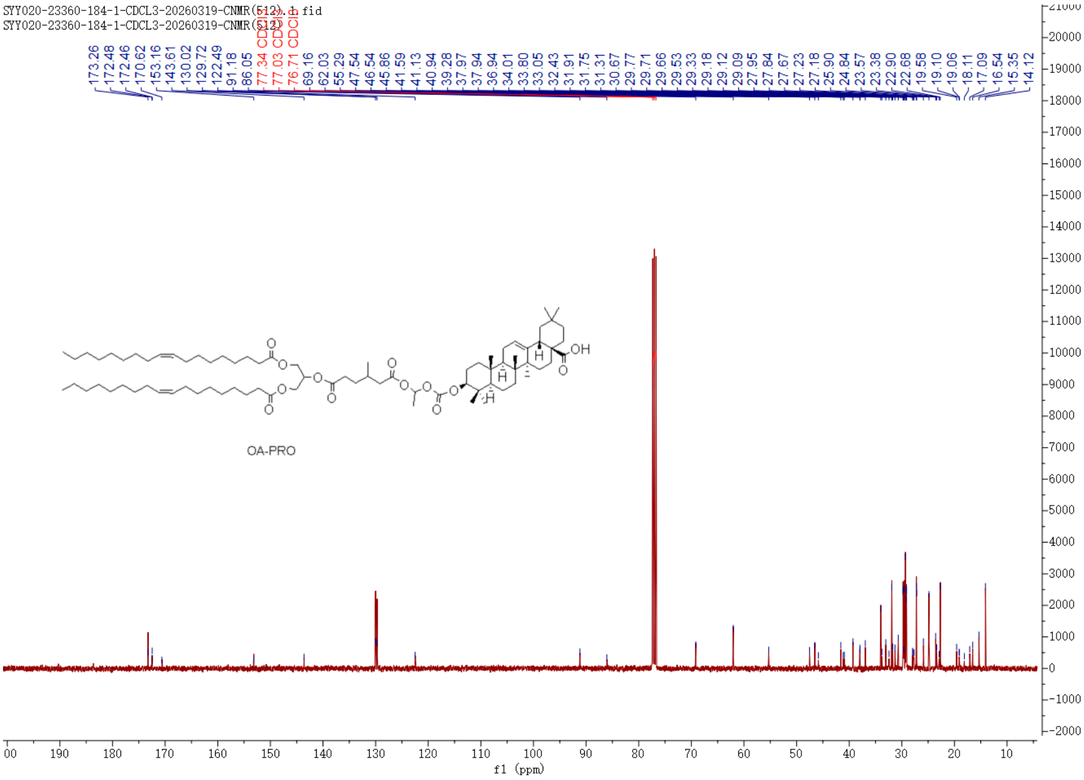

OA-PRO TOF-MS (ESI−): *m/z* 1287.9996 [M − H]−

Sample Chromatograms

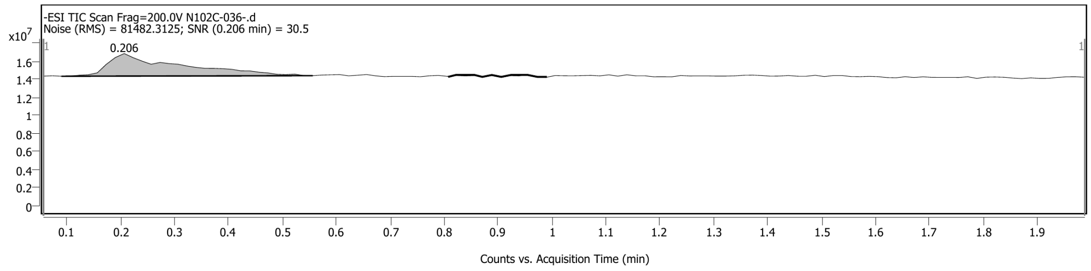

Sample Spectra

- Scan (rt: 0.156-0.473 min) Sub Peak 1 from - TIC Scan

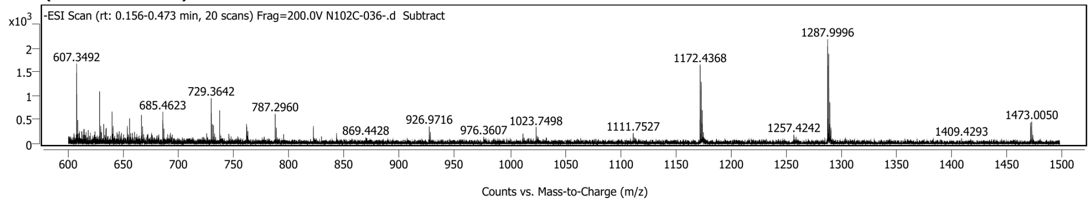

Supplement: Supplementary file 1 [file pharmaceutics-18-00899-s001.zip › pharmaceutics-4420898-supplementary.pdf]
